# Activation of Subcutaneous Mast Cells in Acupuncture Points Triggers Analgesia

**DOI:** 10.3390/cells11050809

**Published:** 2022-02-25

**Authors:** Li-Na Wang, Xue-Zhi Wang, Yu-Jia Li, Bing-Rong Li, Meng Huang, Xiao-Yu Wang, Ryszard Grygorczyk, Guang-Hong Ding, Wolfgang Schwarz

**Affiliations:** 1 School of Acupuncture-Moxibustion and Tuina, Shanghai University of Traditional Chinese Medicine, Shanghai 201203, China; lnwang@shutcm.edu.cn (L.-N.W.); liyujia36@shutcm.edu.cn (Y.-J.L.); 2 Shanghai Key Laboratory of Acupuncture Mechanism and Acupoint Function, Department of Aeronautics and Astronautics, Fudan University, Shanghai 200433, China; xuezhiwang@fudan.edu.cn (X.-Z.W.); brli19@fudan.edu.cn (B.-R.L.); 3 Shanghai Research Center for Acupuncture and Meridians, Shanghai 201203, China; m_huang@fudan.edu.cn; 4 Laboratory of Immunology and Virology, Experimental Center for Science and Technology, Shanghai University of Traditional Chinese Medicine, Shanghai 201203, China; 0000002733@shutcm.edu.cn; 5 Department of Medicine, Université de Montréal, Montréal, QC H3T 1J4, Canada; ryszard.grygorczyk@umontreal.ca; 6 Institute for Biophysics, Department of Physics, Goethe-University Frankfurt, Max-von-Laue St. 1, 60438 Frankfurt am Main, Germany

**Keywords:** mast cells, acupuncture, analgesia, mechanosensitivity, TRPV, purinergic signals, histamine, serotonin

## Abstract

This review summarizes experimental evidence indicating that subcutaneous mast cells are involved in the trigger mechanism of analgesia induced by acupuncture, a traditional oriental therapy, which has gradually become accepted worldwide. The results are essentially based on work from our laboratories. Skin mast cells are present at a high density in acupuncture points where fine needles are inserted and manipulated during acupuncture intervention. Mast cells are sensitive to mechanical stimulation because they express multiple types of mechanosensitive channels, including TRPV1, TRPV2, TRPV4, receptors and chloride channels. Acupuncture manipulation generates force and torque that indirectly activate the mast cells via the collagen network. Subsequently, various mediators, for example, histamine, serotonin, adenosine triphosphate and adenosine, are released from activated mast cells to the interstitial space; they or their downstream products activate the corresponding receptors situated at local nerve terminals of sensory neurons in peripheral ganglia. The analgesic effects are thought to be generated via the reduced electrical activities of the primary sensory neurons. Alternatively, these neurons project such signals to pain-relevant regions in spinal cord and/or higher centers of the brain.

## 1. Introduction

In recent years, acupuncture has become accepted worldwide as an effective therapy, in particular for analgesic therapy [1]. This acceptance is not only based on the effectiveness of acupuncture, but also on the growing understanding of underlying cellular and molecular mechanisms (see also e.g., Wang et al. (2022 in press) [2]). Acupuncture points (acupoints) are characterized by a high density of subcutaneous mast cells (MCs); their activation has turned out to play a crucial role in the initiation of the analgesic effect (see Section 2). The classical way of stimulating acupoints in traditional Chinese medicine (TCM) is the thrusting and twisting of the acupuncture needle or the pressing and rubbing during tuina intervention. As well, application of electrical potential changes (electroacupuncture) in the range of a few tens of volt, or of heat (moxibustion) to the needle are in common use. Recently, laser acupuncture [3] was introduced into TCM treatments by directing visible or infrared (IR) light beams to the acupoints. In our review article, we intend to focus on the mechanisms in Section 3 that lead to the MC activation in response to all those direct acupuncture stimulations, especially mechanical stimulation by acupuncture needling. The activation is based on activation of ion-selective channels leading to an intracellular Ca^2+^ increase and MC degranulation. Various mediators become released during the activation of MCs that are needed for the analgesic acupuncture signaling (see Section 4). The interaction of the MCs with nerve endings is the initiating and essential step in the signaling process, which is based on the mechanisms described in the preceding sections (see Section 5). In Section 6, we will summarize all the processes that lead to the acupuncture effects and those discussed in this review; in addition, prospects will briefly be presented.

## 2. Activation of MCs Contributes to Acupuncture Analgesia

MCs are a kind of multifunctional immune cells that exist in connective tissue and mucosal tissue. When the MCs are stimulated, they degranulate and release different types of biologically active substances. Under normal circumstances, MCs derived from hematopoietic stem cells [4,5] migrate to different locations in the body through blood circulation and perform their physiological functions. According to different tissues where cells are located, rodent MCs can divide into two subgroups, connective tissue mast cells (CTMC) and mucosal mast cells (MMC) [6]. CTMC and MMC are located in different organizations and have different shapes and functions [4].

### 2.1. MCs in Acupoints

For acupuncture effects, the abundance of nerve and vascular systems represent acupoint structures that can serve as putative targets for acupuncture and moxibustion [7,8,9,10]. However, these two fixed structures alone cannot explain all the phenomena and mechanisms of their clinical efficacy [10]. For example, sensitization of the acupoints phenomenon, Ashi point mechanism, delayed response after acupuncture, the broad-spectrum effect of acupuncture and dynamics and movement of the acupoint phenomenon [10,11,12,13]. These phenomena can partially be explained by the residence of subcutaneous MCs in acupoints, due to their biological function, location and density, and relationship with the nervous and vascular system [10].

**Figure 1:**
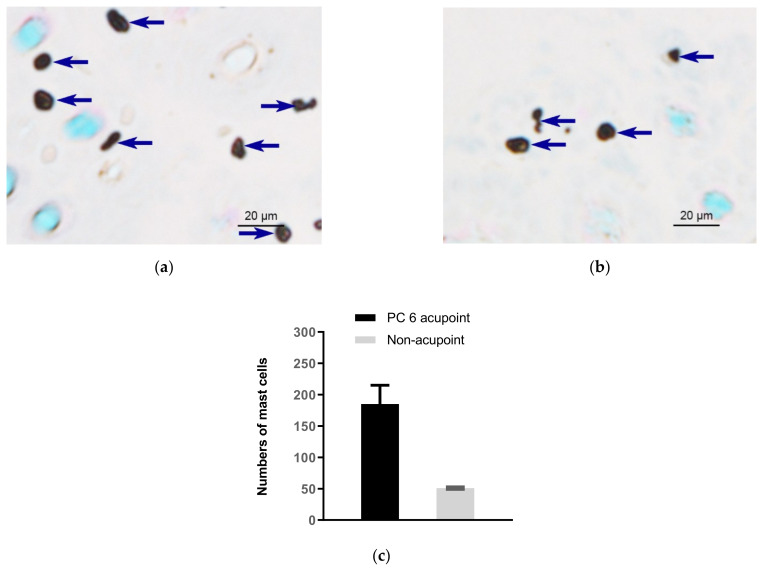
Comparison of subcutaneous MC density in acupoints and non-acupoints of normal rabbits. (**a**,**b**) Paraffin sections prepared from PC6 acupoints and non-acupoints, respectively. MCs were labeled with toluidine blue and shown as dark spots. Dark blue arrows indicate MCs. (**c**) Numbers of MCs in PC6 and the non-acupoint. Data represent means + SEM (*n* = 3), and are based on Zhu et al. (2017) [17], with permission from Springer Nature.

In 1977, Song [14] first proposed that human skin MCs are related to the phenomenon of meridians. When we apply acupuncture, it may stimulate the MCs in the acupoint tissues, causing the cells to degranulate and release biologically active substances such as histamine, serotonin, adenosine triphosphate (ATP) and its metabolic products, and other bioactive substances. These substances have the functions of changing capillary permeability, dilating small blood vessels and contracting smooth muscle cells, thereby “stimulating” local meridian transmission. Subsequently, researchers at the Department of Embryology and Pathology of Liaoning College of Traditional Chinese Medicine [15] observed a significant difference in the total number of MCs in the acupoint area and the non-acupoint area in human amputation specimens. By comparing with the non-acupoint area, they confirmed that the relative number of MCs in the acupoint area was comparably high. Zhu and Xu (1990) [16] observed that the distribution of MCs in humans and rats along the meridian low-resistance line or the meridian sensory line was more than in the control area. Figure 1 shows comparison of subcutaneous MC density in the acupoint (Neiguan acupoint, (PC6)) and in non-acupoint of rabbits [17].

The correlation of MC distribution with the acupuncture systems in humans has been explored in detail by Li (2019) [18]. After examining 285 skin biopsies, dermal MC-enriched special sites are found in the head and limbs, and around orifices of body surfaces. Patterns of MC distribution are highly correlated with the distributions of classic acupoints in 14 classic acupuncture meridians, except for the trunk areas. These findings provide tissue evidence of neuroimmune basis of acupuncture and suggest that MCs mat is a tissue target for acupuncture stimulation and may serve as a tissue marker for acupoints.

**Figure 2:**
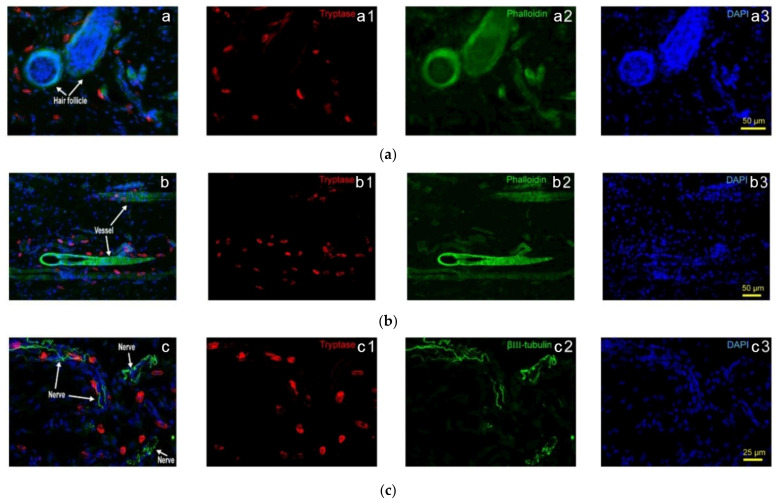
Subcutaneous MC distribution along hair follicles, vessels and nerve fibers of rats. (**a**) Correlation of MCs and hair follicles in skin examined with immunofluorescence histochemical staining with (a1) tryptase (red), (a2) phalloidin (green) and (a3) DAPI (blue). (**b**) Correlation of MCs and vessels in skin samples examined with immunofluorescence histochemical staining with (b1) tryptase (red), (b2) phalloidin (green) and (b3) DAPI (blue). Hair follicles and vessels are distinguished by their location and morphological characteristics. (**c**) Correlation of MCs and hair follicles in skin examined with immunofluorescence histochemical staining with (c1) tryptase (red), (c2) β-III tubulin (green) and (c3) DAPI (blue). Scale bars are shown in the DAPI-stained figure in each group. Based on Yang et al. (2018), [20], with permission from Ivyspring International Publisher.

Closer observations find that in normal rats, Hegu acupoint (LI4), MCs aggregate in close proximity to the blood vessels in intra-epidermis and dermis, and some of them with degranulation in the lower dermis and subcutaneous [19]. Further observations show that rats MCs relatively concentrate around hair follicles and blood vessels, showing distribution characteristics along with hair follicles (Figure 2(a1–a3)) and blood vessels (Figure 2(b1–b3)) (see also Yang et al. (2018) [20]). In addition, the distribution of MCs along the nerve fibers is also relatively concentrated. Although the direction and form of the nerve fibers in the tissue are very irregular, the MCs arrange along with the nerve fibers (Figure 2(c1–c3)) (see also Yang et al. (2018) [20]).

**Figure 3:**
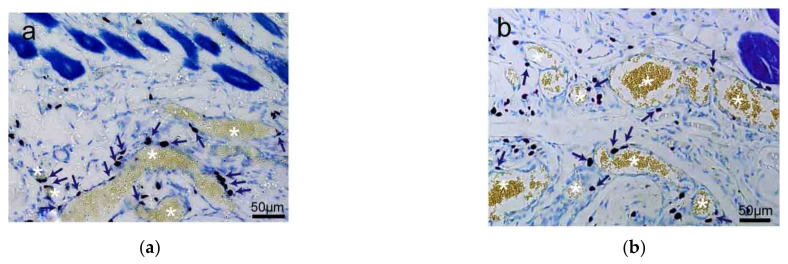

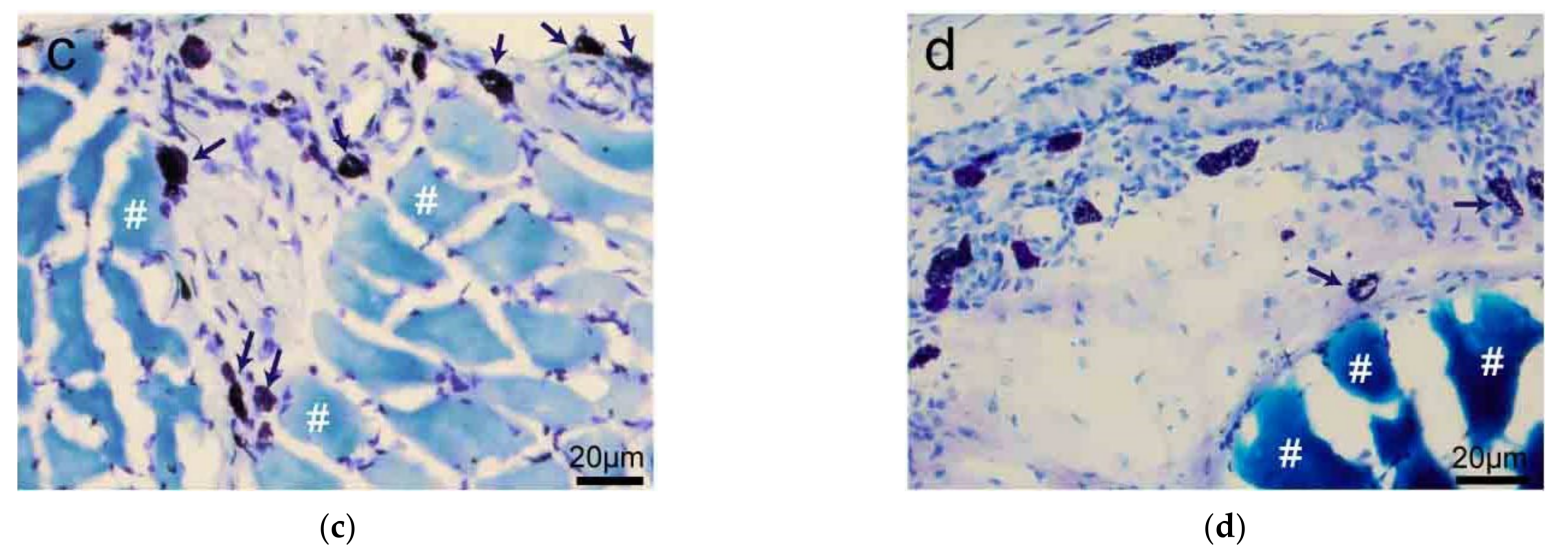
The distribution of rat MCs along adipose and muscle tissues. (**a**,**b**) MCs are distributed along adipose tissue. Adipose tissue is dyed yellow with gathered particles and marked by white asterisks ‘*’ in the figures. (**c**,**d**) MCs are distributed along the muscle tissue. The muscle tissue is dyed blue or dark blue and marked by white pound signs ‘#’ in the figures. MCs around the tissue boundaries are marked with dark blue arrows, and the scale bar is shown in the lower right-hand corner of each examined figure. Based on Yang et al. (2018) [20], with permission from Ivyspring International Publisher.

Besides skin tissue, MCs also distribute along with the adipose tissue (Figure 3a,b) and are unevenly distributed around the edge of muscle tissue (Figure 3c,d) [20].

**Figure 4:**
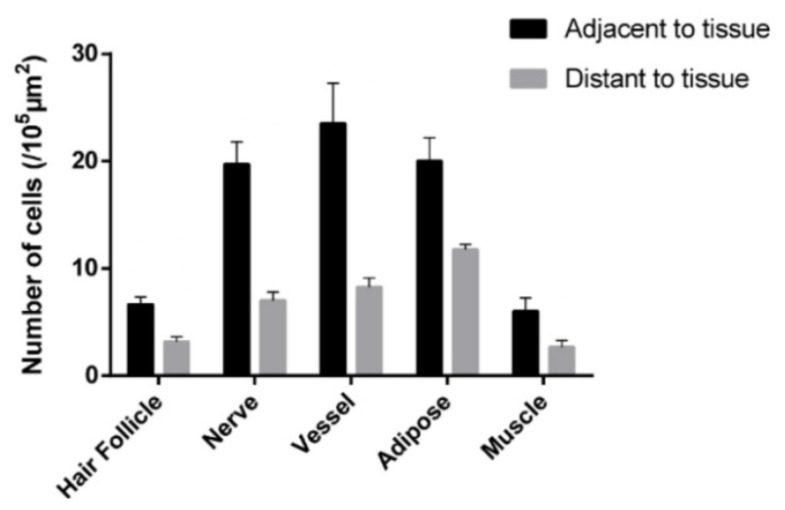
Comparison of the MC number at adjacent and distant to hair follicles, nerves, blood vessels, adipose and skeletal muscles in rats. Data represent means + SEM (*n* = 4–7) and are based on Yang et al. (2018) [20], with permission from Ivyspring International Publisher.

Further statistical analysis reveals that more MCs tend to aggregate around the hair follicles, nerves, blood vessels, adipose and skeletal muscles (Figure 4) [20].

Additionally, MCs tend to aggregate at the junction of different stiffness. In in vitro experiments, rat alkaline leukemia mast cells (RBL-2H3 cell line) are allowed to grow on a piece of polydimethylsiloxane (PDMS) membrane that is spliced with regions of varying hardness. Eventually, RBL-2H3 cells migrate and tend to stay at the site with stiffness changes [20]. Further in vivo tests in rats find that subcutaneous MCs in acupoints distribute spatially unevenly and preferentially distribute in parts of the tissue or extracellular matrix with changes in stiffness [20]. These findings indicate that changing the stiffness of local tissues may lead to the accumulation of MCs. Based on the origin of MCs and experimental results, it can be predicted that MCs exist in tissues containing permeable capillaries and are more inclined to choose areas with changes in stiffness [20].

The increased density of MCs can be understood as an increase in the development and aggregation of MCs. The MC progenitor develops into mature MCs by stem cell factor (SCF) and its receptor c-kit [21]. In addition, local cytokines and/or chemokines can increase MC number. Monocyte chemoattractant protein-1 (MCP-1) is a key chemokine that regulates the migration and infiltration of MCs [22]. It is reported that in normal rats, electroacupuncture (0.1 mA intensity, 2/15 Hz alternating wave, 25 min) at the Weishu acupoint (BL21) and Zusanli acupoint (ST36) increases the local MC number and promotes them to degranulate [23]; such increased MC number rather than degranulation can be prevented by local pre-administration of SCF antibody (anti-SCF) [23]. Subsequent series of experiments reveal that MC number in acupoints is positively correlated to the MCP-1 content [24]. The presence of MCP-1 antibody (anti-MCP-1) in rats BL21 decreases local MC number and degranulation ratio; moreover, pre-administration of anti-MCP-1 in BL21 prevents electroacupuncture-upregulated MC count and degranulation ratio [24]. Furthermore, intercellular adhesion molecule-1 (ICAM-1) allows leukocytes to penetrate the walls of blood vessels and exit in the tissue. An increased expression of ICAM-1 mRNA is observed in ST36 that are treated by electroacupuncture (0.1 mA intensity, 2/15 Hz alternating wave, 25 min) [25].

### 2.2. MCs in Acupuncture-Analgesic Effect

**Figure 5:**
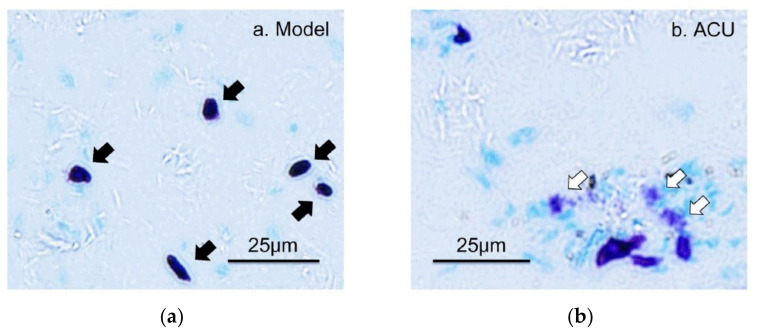

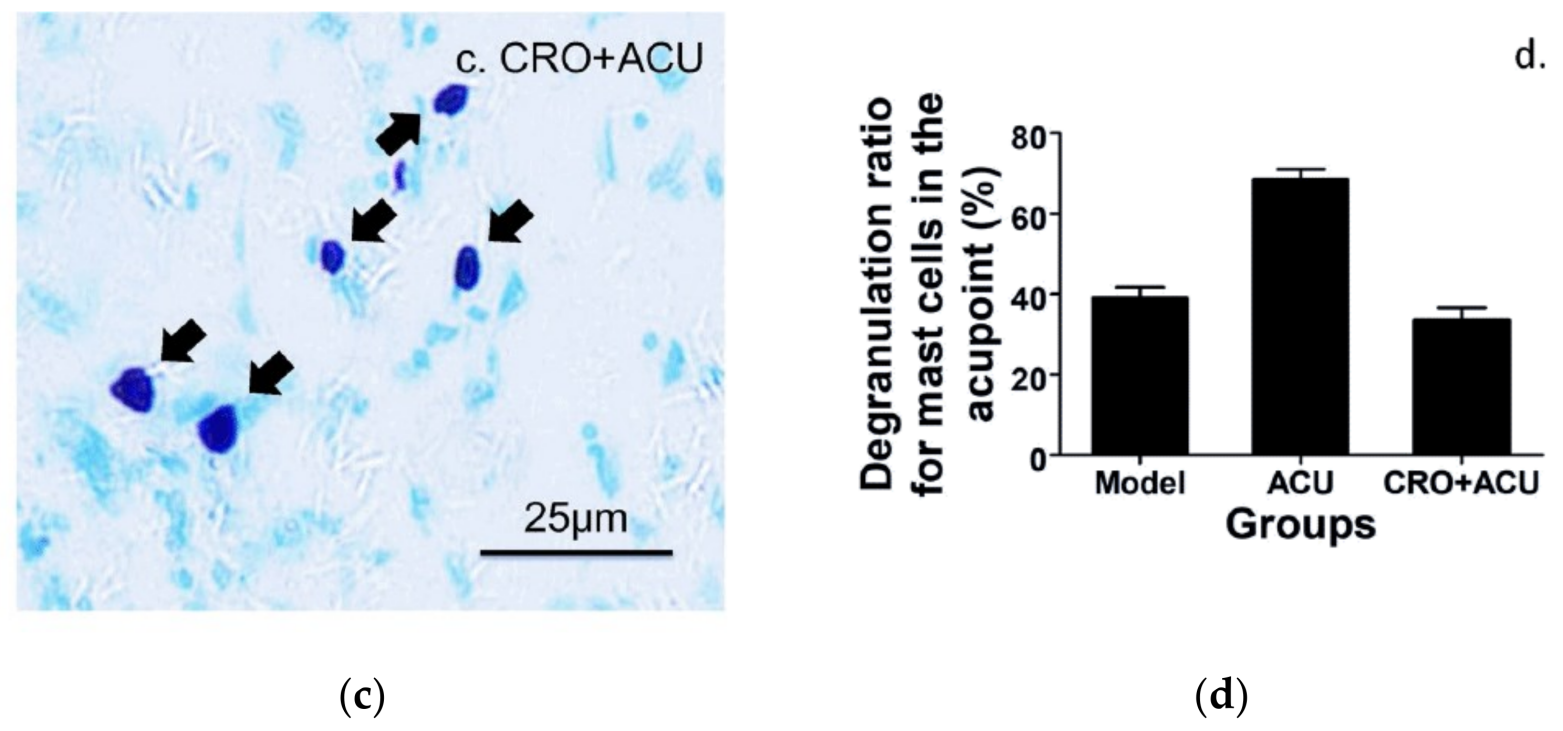
The degranulation of MCs at ST36 induced by acupuncture. (**a**–**c**) Paraffin sections prepared from ST36 of different groups. Inflammation pain was established by injection of 50 μL Complete Freund’s Adjuvant (CFA) into the left ankle joint cavity to induce acute adjuvant arthritis after rat was anesthetized with 1.5% isoflurane. Inflammatory syndrome, local swelling and behavioral disability appeared within 24 h. Two days later, ipsilateral AP treatment (20 min) was performed on left acupoint ST36. Contained MCs were labeled with toluidine blue. CRO (0.02 g/mL, 20 μL) was pre-injected in treated acupoints 20 min before needling. The dark arrows point to intact MCs and the hollow arrows point to degranulated MCs. (**d**) Comparison of the degranulation ratio of MCs in ST36 from different groups. Figure is from Huang et al. (2018) [32],with permission from Springer Nature.

The analgesic effect of acupuncture is produced by needle piercing and manipulation at acupoints. Mechanical stimulation is the main stimulation mode applied to acupoints by needling. Tests in rats reveal that lifting–inserting manipulation generates a force in the range of 240–280 mN, and twisting manipulation produces torque in the range of 10–15 mN × mm^−1^ [26], which is transmitted to the wider and deeper space by subcutaneous collagen fibers twisting around the needles [27,28]. The human body can convert such mechanical stimuli into a recognizable biological signal during acupoint activation, which reaches the target organ after peripheral nerve transmission and central processing to produce a broad-spectrum or specific analgesic effect [2]. The activation of MCs refers to release of granules from the storage of mature MCs, which triggers the biological effects of adjacent cells or tissues. This process is termed degranulation of MCs. There are three main ways of MC activation: immunogenic, neurogenic and physical activation [4,29]. Many studies have confirmed that MCs play a central role in the initiation mechanism of acupuncture analgesic via degranulation [17,30,31,32,33]. In acute adjuvant arthritis (AA) model rats, acupuncture can induce the degranulation of subcutaneous MCs in treated ST36 [30,32], which can be prevented by local pre-administration of cromolyn (CRO), a stabilizer of MCs (Figure 5).

**Figure 6:**
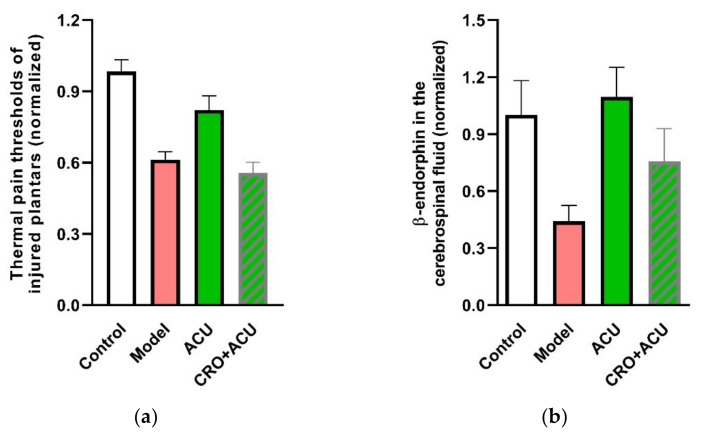
Contribution of MC degranulation to acupuncture analgesia (**a**) and β-endorphin increase in the cerebrospinal fluid. (**b**) Pre-injection of CRO (0.02 g/mL, 20 μL). Establishing of inflammation pain and acupuncture intervention was referred to in the legend of Figure 5. β-endorphin in the cerebrospinal fluid was determined with enzyme linked immunosorbent assay (ELISA). Data represent means + SEM (*n* = 7–14) and are based on Huang et al. (2018) [32].

Moreover, a function test confirm that MC degranulation is an essential step towards an analgesic effect of acupuncture. The ankle AA rats benefit from acupuncture at ST36. However, using CRO to shield the degranulation of MCs in the treated acupoint can significantly weaken the acupuncture analgesic effect [31,32] (Figure 6a). The underlying mechanism might be upregulation of β-endorphin level in the cerebrospinal fluid (Figure 6b) [32].

MC-deficient animals have begun to be used for this issue. Compared with wild-type rats, *c-kit gene* mutant–induced MC-deficient rats (WsRC−+/+ rats) have less MC number in the whole body, including at acupoints, and demonstrate a higher mechanical pain threshold [34]. The critical findings are that in non-painful WsRC−+/+ rats, although acupuncture (10 min, ST36) still can produce an analgesic effect for mechanical pain, the effect is substantially decreased compared with wild-type rats [34]. Besides, WsRC−+/+ rats are no longer sensitive to other TCM treatments. Only noxious stimuli, 46 °C, mimicking moxibustion and 3-mA electroacupuncture, can generate a less pronounced anti-nociceptive effect for mechanical pain [34].

## 3. Activation of MCs by Acupuncture/Physical Stimulations

The manipulation of the acupuncture needle within the connective tissue of acupoints transfers mechanical stress to the imbedded MCs and nerve endings (see Section 2). A crucial step in the initiation of acupuncture effects is activation of the MCs and the exocytotic release of mediators (see Section 4) during the associated degranulation process.

The basis for degranulation is the increase of intracellular Ca^2+^ activity [35], which can partially be attributed to entry of extracellular Ca^2+^ mediated by Ca^2+^-permeable ion channels (Section 3.1 and piezo channels) and to other channels (Section 3.2), which depolarize the membrane potential and facilitate Ca^2+^ entry. These channels can be activated by various physical stimuli, and hence, are likely candidates to play a crucial role in inducing the analgesic acupuncture effect.

### 3.1. TRPV Channels

Vallinoid-sensitive transient receptor potential (TRPV) [36] channels are Ca^2+^-permeable and are expressed in MCs (see e.g., Bradding et al. (2003) [37]). Among the TRPV family the isoforms TRPV1, TRPV2 and TRPV4 are expressed in MCs, which has been demonstrated by Western blot analysis as well as conventional reverse transcription [38].

These TRPV ion channels can be activated by physical stimuli that are used in TCM treatment, including conventional mechanical stimulation as used in the needling acupuncture and tuina, electrical stimulation as used in electroacupuncture, high temperatures as used in application of moxibustion and laser light as used in laser acupuncture (Wang et al. (2022) [2].

The channels also play a crucial role in various pain sensations, and acupuncture can affect the degree of their expression [39,40,41].

#### 3.1.1. TRPV1

TRPV1 was first described by Caterina et al. (1997) [42] as a capsaicin- and heat-sensitive ion channel that allows Ca^2+^ permeation; the discovery was honored by Nobel prize to David Julius in 2021. It was shown that TRPV1 is involved in pain signaling activities [43,44,45], and hence has become an important target for analgesic agents, including the physical stimulation of acupoints. It is characteristically activated by temperatures exceeding 43 °C that is reached within the treated skin area during moxibustion [2,46].

Not only thermal, but also mechanical hyperalgesia has been demonstrated to be involved in activation of TRPV1 [47]. Whole-cell patch-clamp experiments on primary neurons have revealed that changes in the osmolarity of extracellular medium as well as pressures by applying suction to the patch pipette results in an increase of TRPV1-mediated current [47]. Even laser light, used in laser acupuncture, can activate the TRPV1 ion channels; this is demonstrated for green laser light (406 nm) applied to *Xenopus* oocytes with heterologously expressed TRPV1 [48] and for red laser light (640 nm) applied to human MC line, HMC-1 cells [39].A temperature increase to 43 °C, indeed, results in an intracellular increase of Ca^2+^ activity that can be attenuated by chelating extracellular Ca^2+^ by EGTA [46].

#### 3.1.2. TRPV2

**Figure 7:**
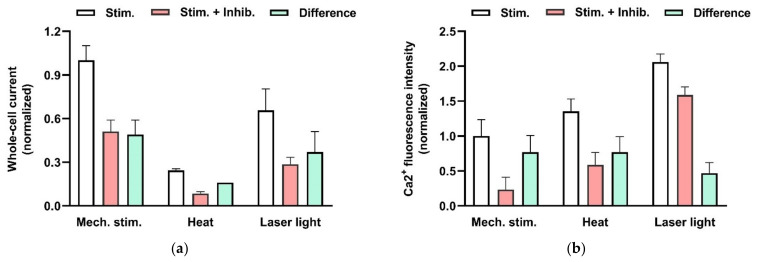
Effects of physical stimulation of HMC-1 on whole-cell patch-clamp current (**a**) and intracellular Ca^2+^ activity (**b**) mediated by TRPV2 channels. (**a**) Currents were recorded at −100 mV and activated by mechanical stress (−60 cm H_2_O) applied to the patch pipette, by heat (exposure to solution preheated to 53 °C), or by red laser light (at 640 nm and 48 mW). The columns represent the current in the absence (not filled) and the presence (red) of an TRPV inhibitor (20 µM SKF96365 or 10 µM Ruthenium red). In addition, the inhibitor-sensitive, TRPV2-mediated components (difference) are shown (green). The data are normalized to the current signal that can be induced by physical stimuli. The signal changes are significant based on *p*< 0.05. Data are extracted from Zhang et al. (2012) [38]. (**b**) Increase in intracellular Ca^2+^ fluorescence intensity was measure in HMC-1 cells loaded with 4 µM “Calcium Green-1AM”. TRPV2 is activated by mechanical stress (hypotonic solution (240 mOsm compared with 310 mOsm) applied to the cell suspension), by heat (exposure to solution preheated to 53 °C), or by red laser light (at 640 nm and 48 mW). The columns represent the intensities in the absence (not filled) and the presence (red) of an TRPV4 inhibitor (20 µM SKF96365). The TRPV2-mediated Ca^2+^ increase is considered to the difference (green). The data are normalized to the fluorescence signal that can be induced mechanically. The signal changes are significant based on *p*< 0.01, and on data from Zhang et al. (2012) [38].

TRPV2 is also selectively permeable for divalent cations [49], allowing Ca^2+^ to enter the cell, and it is characterized by its sensitivity to noxious temperatures exceeding 52 °C [50]. In addition, mechanical stress activates TRPV2. Figure 7a illustrates in whole-cell patch-clamp experiments on the human MCs (HMC-1 cell line) that reduction of intracellular pressure by −60 cm H_2_O, applied via the patch pipette, results in the activation of a current component that is sensitive to the TRPV2-specific inhibitor SKF96365 [38]. Moreover a temperature increase to 53 °C as well as red laser light result in activation of TRPV2 (Figure 7a, see also Zhang et al. (2012) [38]). Figure 7b demonstrates that these stimuli indeed result in increase of intracellular Ca^2+^ that is mediated by activation of TRPV2 (see also Zhang et al. (2012) [38]). Activation of TRPV2 by all the above-mentioned physical stimuli leads to degranulation of MCs ([38,51]).

**Figure 8:**
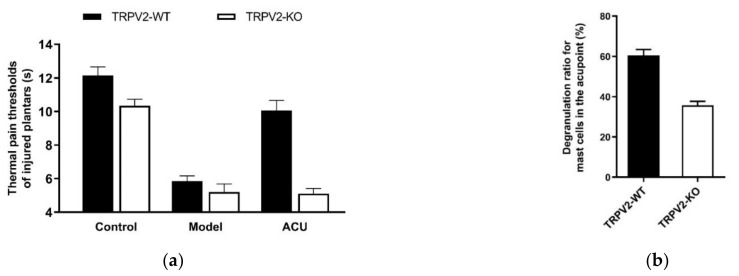
Effects of TRPV2 knock-out on acupuncture analgesia (**a**) and local MC degranulation (**b**) in ankle AA mice. Establishing the inflammation pain and acupuncture intervention was referred to in the legend of Figure 5. In (**a**), data show pain thresholds of injured plantars in response to heat (thermal hyperalgesia) stimulation. In (**b**), data are obtained from the paraffin sections are prepared from ST36 of wild-type (TRPV2-WT) and TRPV2 deficient (TRPV2-KO) mice. MCs were labeled with toluidine blue and were manually counted under microscope. Data represent means + SEM (*n* = 10–11). Figure is from Huang et al. (2018) [32] with kind permission from Springer Nature.

In vivo testing with TRPV2-deficient mice further reveals that compared with wild-type mice, TRPV2-KO mice with ankle inflammation have null response to acupuncture (Figure 8a), which is accompanied by less MC degranulation (Figure 8b) [32].

Together, these findings imply that MC-associated TRPV2 channels may contribute to trigger mechanism of acupuncture analgesia by mediating mechanosensitive degranulation of MCs.

#### 3.1.3. TRPV4

**Figure 9:**
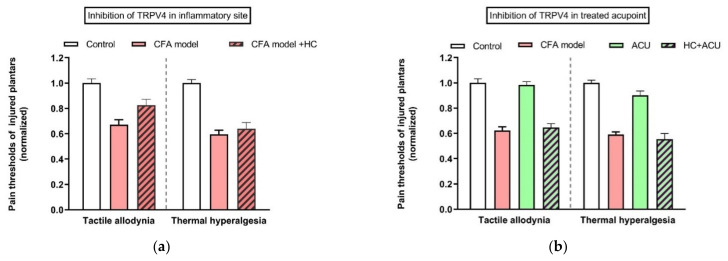
Comparison of TRPV4 channel function at sites of inflammation (**a**) and the treated acupoint (**b**) of ankle AA rats. Establishing the inflammation pain and acupuncture intervention was referred to in the legend of Figure 5. Data show pain thresholds of injured plantars in response to mechanical (tactile allodynia) or heat (thermal hyperalgesia) stimulation. HC067047 (0.2 mM, 20 μL) (HC), a specific inhibitor of TRPV4, was administrated in the injured plantar 50 min before behavioral test (**a**), or in the treated ST36 20 min before 30-min needling. (**b**) Data represent means + SEM (*n* = 6–9), and are based on data from Zheng et al. (2021) [54].

In contrast to TRPV1 and 2, TRPV4 is not a thermo receptor [52]. The main characteristic of TRPV4 is its sensitivity to mechanical stress [53], which also makes TRPV4 a candidate to being activated by acupuncture [54]. Besides MCs (see above), TRPV4 mRNA also presents in the skin tissue of rat acupoints, rather than in the skeletal muscle layer [54]. TRPV4 channels are mostly considered as pain sensors [55,56] to maintain mechanical allodynia via its mechanosensitivity [57]. In line with these findings, pharmacological inhibition of TRPV4 channels at the inflammatory site can relieve tactile allodynia, but not thermal hyperalgesia (Figure 9a) [54]. Interestingly, blockage of TRPV4 channels at the treated ST36 acupoint prevents acupuncture intervention to play anti-nociceptive function (Figure 9b) [54].

In *Xenopus* oocytes, with heterologously expressed TRPV4 ion channels in addition to mechanical stress, green laser light (532 nm) can activate the TRPV4-mediated current [58]. In RBL-2H3 cells, blue (405) or green (532) laser light leads to histamine release, which is attributed to activation of TRPV4 channels [59].

**Figure 10:**
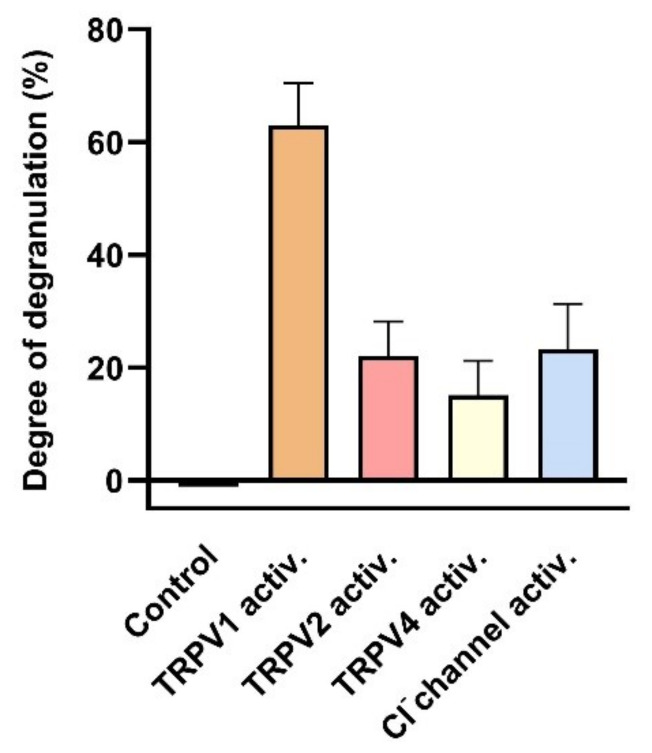
Degranulation of human MCs induced by activation of TRPV1, TRPV2, TRPV4 and stretch-activated (SA) Cl^−^ channels. Degranulation was determined as degree of degranulation induced by the TRPV1 activator capsaicin (1 µM) (data extracted from Gu et al. (2012) [48]), or as the contribution sensitive to specific inhibitors: 20 µM SKF96365 for TRPV2 to degranulation induced by hypotonic stress (230 compared with310 mOsm/L) (data extracted from (Wang and Schwarz (2012) [60]), or 500 nM HC067047 for TRPV4 to degranulation induced by compound 48/80 (data extracted from Mascarenhans et al. (2017) [61]), or 200 µM DIDS for the mechanical SA Cl^−^ channel (see Section 3.1.2) (data extracted from Wang and Schwarz (2012) [60]). Data represent averages + SEM.

Indeed, activation of TRPV1, TRPV2 and TRPV4 channels leads to MC degranulation (see Figure 10). Activation of TRPV1 by its specific agonist capsaicin results in an increase of cell degranulation of HMC-1 cells to more than 60% [48]. Furthermore, MC degranulation induced by hypotonic stress was attenuated in the presence of the TRPV2-specific antagonist SKF96365 [60]. Degranulation induced by the activating compound 48/80 is reported to be attenuated by the TRPV4-specific antagonist HC067047 [61].

In conclusion, the activation of TRPV1, TRPV2 as well as TRPV4 contributes to the MC activation induced by various physical stimuli used in the various treatments in TCM. The associated degranulation contributes as an initiating step to the analgesic effect.

### 3.2. Chloride Channels

**Figure 11:**
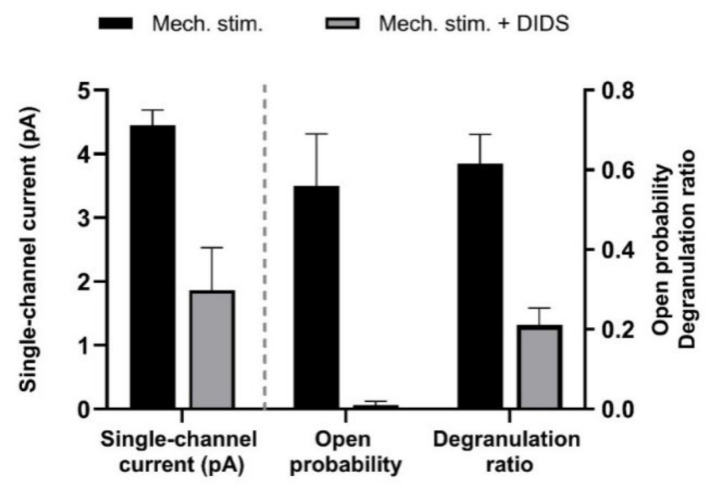
Effect of mechanical stress on single-channel activity and degranulation ratio of human MCs (HMC-1 cell line). Channel activity was induced in outside-out membrane patches by applying negative pressure to the pipette (−40 cm H_2_O); the test potential was + 80 mV. 200 µM DIDS reduced single-channel-current amplitude as well as open-state probability. Degranulation was observed under inverted light microscope. Data represent means + SEM (*n* = 3–4), and are based on Wang et al. (2010) [62].

Mechanical stress can also activate stretch-activated chloride channels (SACs) that can be identified in MCs by their sensitivity to 4, 4′-Diisothiocyano-2, 2′-stilbenedisulfonic acid (DIDS). Hypotonic solution activated in HMC-1 cells as well as MCs from rat tissue is an outward rectifying current component that is partially sensitive to DIDS [60]. Similar results were obtained by applying negative pressure to the patch pipette in the whole-cell mode (see Figure 11). The activation of the SACs also leads to elevation of [Ca^2+^]_i_ as measured by fluorescence technique [60,62]. Consequently, the MCs respond with DIDS-sensitive degranulation (Figure 11).

## 4. Mediators of Acupuncture Signaling

### 4.1. Adenosine and ATP

Extracellular ATP (eATP) and other purine and pyrimidine compounds, such as ADP, adenosine (Ado), UTP, UDP and UDP-glucose, are important mediators in purinergic signaling. They are released from cells in the tissue in response to a variety of physical stimuli, especially mechanical stresses, such as stretch, deformation and shear stress [63,64]. Once released, extracellular nucleotides modulate numerous patho/physiological processes by activating purinergic receptors on target cells, which include ionotropic (P2X) and G protein-coupled (P2Y and P1) receptors. While extracellular nucleotides function as a primary messenger in intercellular communication, the range of their effects can vastly be expanded by stimulating the release of other extracellular messenger substances, including neurotransmitters, hormones, growth factors, enzymes, cytokines, lipid mediators, nitric oxide and reactive oxygen species [65].

**Figure 12:**
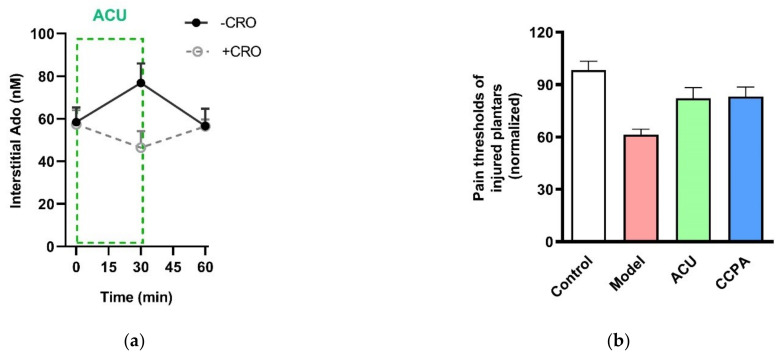
Contribution of Ado and A1 receptors to acupuncture analgesia. (**a**) Extracellular Ado level in response to 30-min needing in the absence and presence of CRO. Ado was collected with microdialysis method for each 30 min and assayed with high-performance liquid chromatography. CRO (0.02 g/mL, 20 μL) was pre-injected in treated acupoints of rats 5 min before needling. (**b**) Changes of thermal pain thresholds of the injured plantars in response to acupuncture or activation of A1 receptors. 2-Chloro-*N*6-cyclopentyladenosine (CCPA) (0.04 mg/mL, 20 μL), a specific agonist of A1 receptors, was injected in acupoint rats 30 min before behavioral tests. Establishing the inflammation pain and acupuncture intervention was referred to the legend of Figure 5. Data represent means + SEM (*n* = 7–11) and are based on Huang et al. (2018) [32].

In 2009, Burnstock [66] hypothesized that beneficial effects of acupuncture may involve triggering the purinergic signaling cascade via ATP release in acupoints. This notion intensified research on the role of purinergic signaling, especially P2 receptors of the nerve system in acupuncture [67]. It has been evidenced that needling promotes extracellular adenosine (Ado) accumulation in treated acupoints of humans [68] and rodents [32,69]. In healthy male volunteers, 30-min acupuncture increases interstitial Ado concentration in the treated ST36 [68]. In ankle AA mice, Ado, together with AMP, ADP and ATP, aggregates in the treated ST36 in response to 30-min acupuncture [69]. Further study in ankle AA rats reveals that Ado accumulation in the needling-treated ST36 can be canceled by preventing MC degranulation with CRO administration (Figure 12a), which is accompanied by the suppression of the acupuncture analgesic effect, as mentioned above (Figure 6). Besides manual acupuncture, electroacupuncture also can mediate Ado aggregation. In pituitrin-induced acute heart bradycardia rabbits, Ado accumulation occurs in Neiguan point (PC6) treated by 30-min electroacupuncture [33]. In ankle inflammatory or neuropathic pain model mice, activation of Ado A1 receptors in acupoint ST36 by 2-Chloro-*N*6-cyclopentyladenosine (CCPA), a specific agonist of A1 receptors, has an anti-nociceptive effect [69]. Similarly, in ankle AA rats, injection of CCPA in acupoint ST36 can duplicate needling-induced anti-nociception (Figure 12b) [32]. While once A1 receptors are knockout, acupuncture no longer functions [69].

The aggregated Ado can originate from the activated MCs [70] or be converted from other co-released nucleotides (such as ATP and ADP). The latter cannot be ignored because ATP is a stress-responsive molecule in various mammalian cells [63], and mechanical force is the main stimulus generated by needling in acupoints [71], as mentioned in Section 2.2. It is entirely possible that acupuncture can facilitate ATP release from subcutaneous MCs that are mechanosensitive, as mentioned in Section 3.

**Figure 13:**
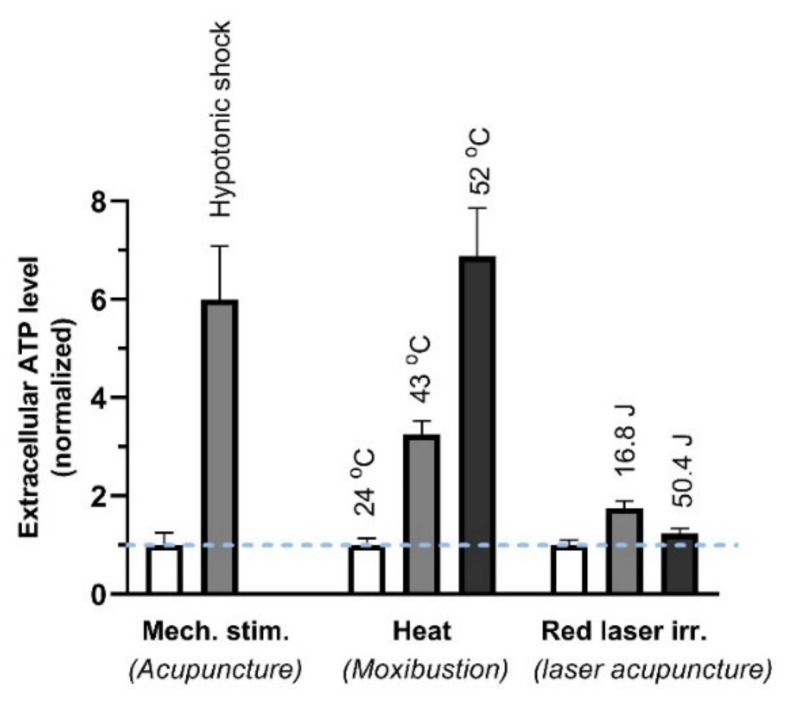
ATP released from MCs (HMC-1 cell line) in response to different stimuli. The cell suspension was perturbed by 3-min, 50% hypotonic shock (of acupuncture), 3-min heat (of moxibustion) or irradiation of 657-nm laser irradiation (280 mW/cm^2^) (of red laser acupuncture). Energy density (J/cm^2^) = power density (W/cm^2^) × time (s). The data represent averages + SEM (*n* = 4–16) and are based on Wang et al. (2013) and Wang et al. (2015) [46,73].

ATP has been confirmed in vitro to be released from MCs by various physical stimuli (Figure 13) that are performed to mimic some external TCM treatments, including needling acupuncture, moxibustion and laser light [72].

**Figure 14:**
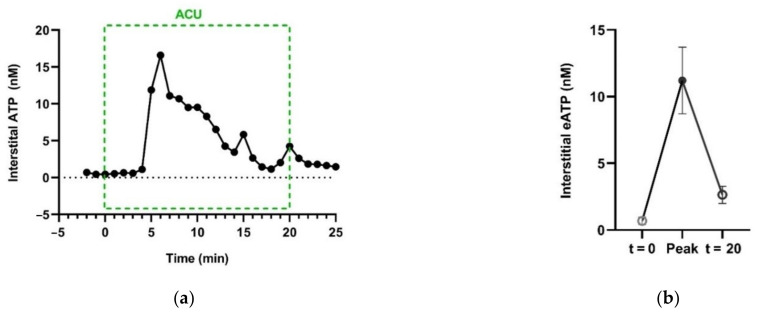
Acupuncture-induced transient accumulation of interstitial ATP in treated acupoints. (**a**), Representative trace of ATP concentration in response to needling manipulation, dominated by twirling-rotating (~100 times/min), supplemented with lifting-thrusting (~80 times/min). ATP in the interstitial space was collected with microdialysis method and assayed with luciferase-luciferin assay. (**b**) Comparison of [eATP] of baseline (t = 0), peak and termination of AP. See Figure 5 legend for the establishment of the inflammation pain and acupuncture intervention. The data represent averages + SEM (*n* = 4) and are based on data from Zuo et al. (2022) [74].

Additionally, in vivo, needling manipulation causes eATP to transiently accumulate in the interstitial space of the treated ST36 of ankle AA rats (Figure 14) [74].

**Figure 15:**
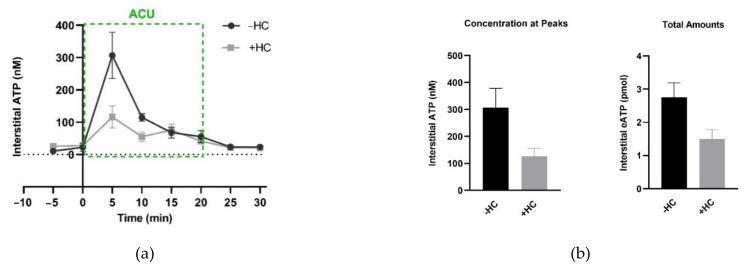
Effect of TRPV4 channel inhibition at the acupoints on needling-induced eATP accumulation. (**a**) Time course of eATP changes during AP in the absence and presence of HC067047 (HC) (0.2 mM, 20 μL), a specific antagonist of TRPV4. Each sample contained 5-min microdialysis. (**b**) Comparison of eATP peaks and the total amounts, respectively, in the absence and presence of HC067047 (0.2 mM, 20 μL). Graphs are based on the same data as (**a**); eATP amounts were calculated for the area under the curve from 0 min to 20 min. The data represent averages + SEM (*n* = 4–5). Figure is from Zheng et al. (2021) [54] with kind permission from MDPI.

Both nonlytic release and cell lysis can lead to such interstitial ATP aggregation during acupuncture. Nonlytic release of ATP can be mediated by some mechanosensitive channels, for example, Piezo channels [75,76,77] and TRPA1 channels [78], and also including TRPV4 channels [63,78], mentioned in Section 3.1.3. In ankle AA rats, pharmacological inhibition of TRPV4 channels in treated ST36 in advance can suppress acupuncture-induced eATP increase (Figure 15), which is accompanied by reduced analgesic effect, as we demonstrated in Figure 9b [54].

It is reported that damage-associated molecules, high mobility group box 1 protein (HMGB1) and toll-like receptor 4 (TLR4), increases in ST36 of normal rats treated by 2-min needling [79], implying the occurrence of tissue damage caused by needle piercing and subsequent manipulation. Thus, ATP release due to cell lysis is also involved in this process.

**Figure 16:**
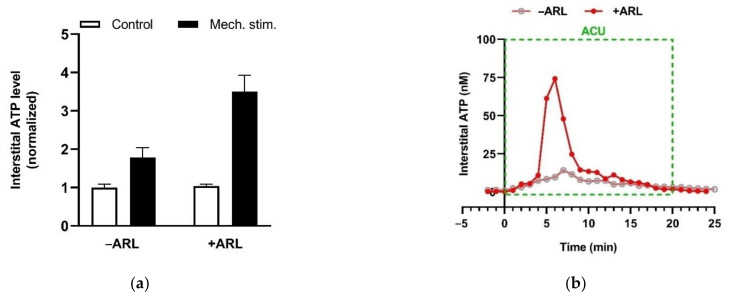
Ecto-nucleotidase activities in MCs in vitro and in rats acupoint in vivo. (**a**) Mechanosensitive ATP release from rat MCs (RBL-2H3 cell line) (averages + SEM, *n* = 4). Medium displacement-induced ATP release was measured in the absence or presence of ARL67156 (100 μM) (*n* = 6–11). ARL67156 was pre-introduced to cell suspension 20 min before mechanical stimulation. (**b**) Potentiating effect of ecto-nucleotidase inhibition on acupuncture-induced eATP accumulation in ST36 of ankle AA rats. Representative trace of acupuncture-induced eATP accumulation in the absence or presence of ARL67156 (100 μM), a non-specific antagonist of ecto-nucleotidases, in the microdialysis solution (*n* = 4). Establishing the inflammation pain and acupuncture intervention was referred to the legend of Figure 5. The data are based on Wang et al. 2020 [83] and Zuo et al. 2022 [74].

eATP is generally accepted as a proinflammatory mediator, and can enhance pain perception via activation purinergic receptors (P2 receptors) [80]. Free ATP (1 or 10 mM) iontophoresed into the skin generates a moderate burning pain in humans [81]. Similarly, free ATP (1 μM) injected into a rat’s hindpaw also elicits a nocifensive response [82]. However, the presence of ecto-nucleotidases may reverse eATP role in the pain mechanism via promoting Ado production. Preventing ATP hydrolysis by ARL67156, a non-specific antagonist of ecto-nucleotidases, potentiates the mechanosensitive ATP release from HMC-1 cells in vitro (Figure 16a) and eATP accumulation in needling-treated acupoint in vivo (Figure 16b), indicating the expression of ecto-nucleotidases in MCs and acupoints.

Rt-PCR determination in rats’ acupoints confirms the presences of multiple types of ecto-nucleotidases [54,74]. Among them, NTPDase 1 and Nt5e are the major nucleotide-hydrolyzing enzymes, which degrades ATP to AMP with intermediate of ADP and hydrolyzes AMP into Ado, respectively [84].

**Figure 17:**
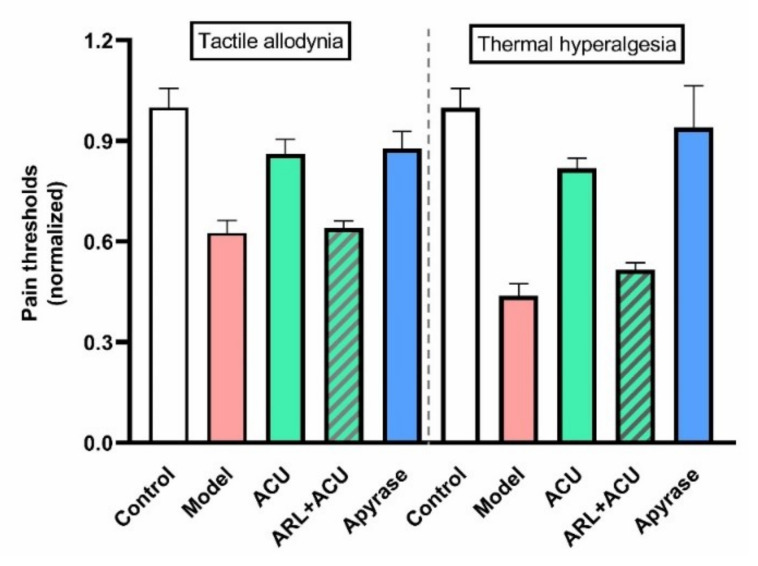
Effect of ecto-nucleotidases’ activities on acupuncture analgesia of ankle AA rats. ARL (100 μM, 50 μL) was pre-injected in ST36 20 min ahead of needling (*n* = 4–5). Apyrase (50 units/mL, 50 μL), soluble form of CD 39, was administrated at the same time point, 50 min before the behavioral test. Establishing the inflammation pain and acupuncture intervention was referred to in Figure 5. The data are based on Zuo et al. 2022 [74].

Further study in ankle AA rats reports that up- and downregulating eATP levels in treated ST36 can dampen and duplicate acupuncture analgesic effect, respectively (Figure 17), which further implies the contribution of ATP mobilization, including release and hydrolysis, to acupuncture analgesia.

**Figure 18:**
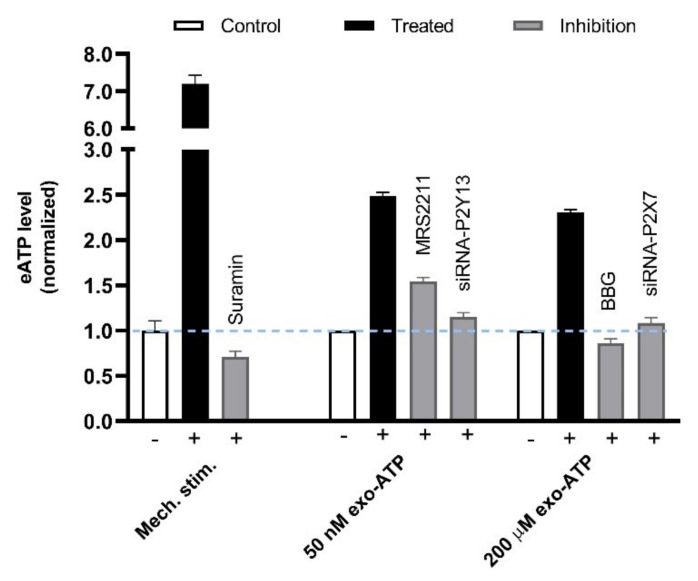
P2Y_13_/P2X_7_ receptors-mediated secondary ATP release from MCs in response to mechanical stimulation. (Left columns), Rat MCs (RBL-2H3 cell line) were irritated by medium displacement that was controlled by electric pipette gun resulting in 6-folds elevation of ATP released to the extracellular space. Such mechanosensitive ATP release could be dampened by the presence of Suramin (100 μM) that was pre-introduced to cells 20 min before stimulation. (Middle columns), 50 nM exogenous ATP (exo-ATP) triggered endogenous ATP release. Exo-ATP was introduced to cells and contained eATP level was assayed immediately. MRS2211 (*n* = 13), a specific antagonist of P2Y_13_, or P2Y_13_ gene interference (*n* = 4) was used to suppress 50 nM exo-ATP-mediated secondary ATP release. (Right columns), 200 μM exo-ATP triggered endogenous ATP release. BBG (*n* = 4), a specific antagonist of P2X_7_, or P2X_7_ gene interference (*n* = 4) was used to suppress 200 μM exo-ATP-mediated secondary ATP release. Data are means + SEM (*n* = 3–11) and are based on Shen et al. (2020) [83].

Besides being the precursor of Ado, ATP released from MCs can function in amplifying the needling signals. Acupuncture-generated acoustic shear waves cause [Ca^2+^]_i_ shock and Ca^2+^ wave propagation (CWP) in vitro and in vivo [85], which indicates the amplification and transmission of localized acupuncture signals. A similar CWP phenomenon occurs to MCs in vitro in response to mechanical stimulation, which can be attributed to ATP-induced ATP release mediated by P2Y_13_ and P2X_7_ receptors [83] (Figure 18).

Clearly, aside from subcutaneous MCs, likely other cell types may also contribute to Ado or ATP aggregation in the treated acupoints. As mentioned above, cell lysis is one of the causes for the aggregation of interstitial ATP during needling. Besides lysis release, other cell types in acupoints, e.g., keratinocytes [86] and skeletal muscle fibers [87], also have properties of mechanosensitive ATP release.

### 4.2. Histamine

Histamine is mainly produced by and stored in MCs. A typical MC in rat connective tissue contains approximately 10–20pg histamine [88]. Once having left the cells, histamine quickly degrades by either histamine *N*-methyltransferase HMT or Deamindiamine oxidase. Half metabolic time of histamine is only about 1 min once released to tissue, which limits the spatial and temporal range of the histamine effect [88].

**Figure 19:**
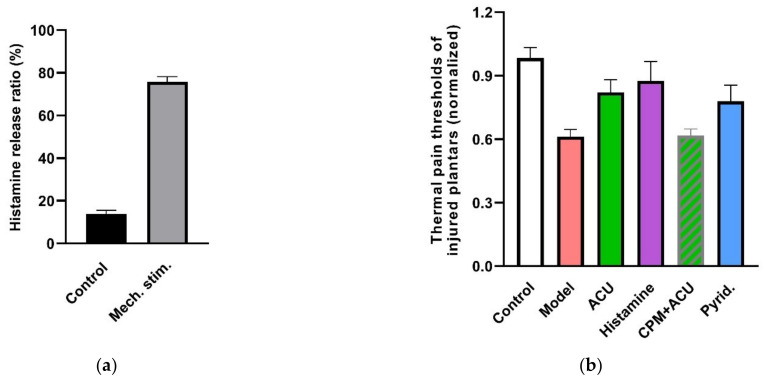

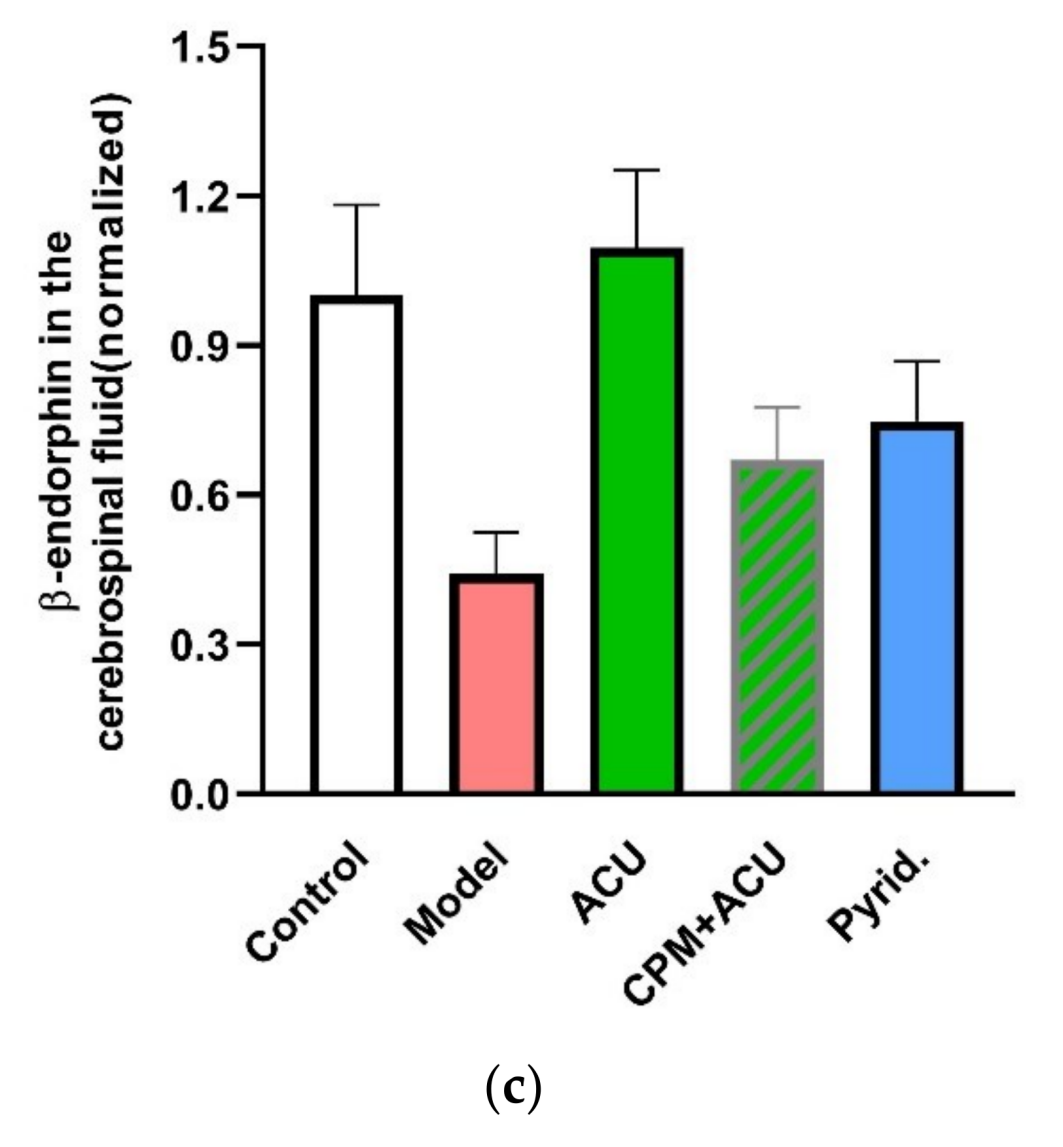
Contribution of histamine and histamine H1 receptors to acupuncture analgesia. (**a**) Mechanosensitive histamine release from HMC-1 cells in response to 240 mOsm-hypotonic shock. Fluorescence intensity in cell supernatants and lyses solutions was determined by fluorescence spectrometer (Hitachi, F-4500) (λex = 350 nm, λem = 440 nm). Released histamine was calculated as the ratio of fluorescences (*n* = 3). (**b**), Effect of histamine and H1 receptors on acupuncture analgesia (*n* = 10–13). (**c**) Effect of histamine H1 receptors on β-endorphin level in the cerebrospinal fluid (*n* = 7–10). CPM (0.4 mg/mL, 50 μL), a specific antagonist of H1 receptors, was pre-injected in ST36 5 min ahead of needling (*n* = 10). Histamine (100 μL/mL, 50 μL) or 2-pyridineethanamine dihydrochloride (Pyrid.) (200 μg/mL, 50 μL), a specific agonist of H1 receptors, was injected at the same time point (*n* = 10). β-endorphin was determined with ELISA. Establishing the inflammation pain and acupuncture intervention was referred to in the legend of Figure 5. Data are means + SEM and are based on Huang et al. (2010) [89], Zhang et al. (2012) [38] and Huang et al. (2018) [32].

Mechanosensitive histamine release is found in HMC-1 cells in vitro (Figure 19a) [38]. In the LI4 acupoint of normal rats, immunohistochemistry analysis finds that histamine is weakly expressed on MCs and becomes strongly expressed on MC, as well as on their separated granules after 3-min acupuncture treatment [19]. Moreover, local administration of histamine alone in ST36 exerts an anti-nociceptive effect in ankle AA rats similar to that of an acupunctured dose (Figure 19b) [89]. Similar findings have been observed by Vieira et al. (2018) [90], whereby local administration of histamine (20 μL, 0.03, 0.3, 3 or 30 mg) induced a dose-dependent analgesic effect and CFA-induced ankle inflammatory and chronic constriction injury (CCI) neuropathic pain in mice.

There are four types of histamine receptors. Among them, notably, the histamine H1 receptor is the interaction target of histamine in the peripheral tissues for multiple responses [91]. In ankle AA rats, an acupuncture analgesic effect can be duplicated by local activation of H1 receptors with 2-pyridineethanamine dihydrochloride (Pyrid.) and can be greatly attenuated by local inhibition of the H1 receptor with chlorprophenpyridamine maleate (CPM) (Figure 19b). Further exploration reveals that modulation of β-endorphin in the central nervous system is its downstream event (Figure 19c).

These findings indicate that MC-associated histamine plays a key role in the trigger mechanism of acupuncture analgesia via activating local histamine H1 receptors.

In addition to playing a role in the initiation mechanisms of acupuncture, histamine in acupoints is also involved in other related events. It can reflect the state of acupoints, whether sensitized or not. For example, histamine-immunopositive MCs becomes more present in the sensitized Yanglinquan acupoint (GB34) and Heding acupoint (EX-LE2) of mono-iodoacetate-induced knee OA rats [92]. In ovalbumin-induced asthma rats, histamine content in the Feishu acupoint (BL13) increases [93]. Beside acupuncture, moxibustion can also modulate local histamine level. In normal rats, 10-min moxibustion at GB34 increases local histamine expression examined by the immunohistochemistry method [94]. In contrast, in asthma rats, the increased histamine content in BL13 has been found to be restored by 14-day moxibustion intervention [93].

### 4.3. Serotonin (5-Hydroxytryptamine, 5-HT)

Serotonin is an another important mediator of MCs [95,96], which plays a potential role in the interaction between MCs and nerve endings [97]. In mono-iodoacetate-induced knee OA rats, serotonin together with tryptase and histamine are upregulated in the sensitized acupoints, GB34 and EX-LE2 [92], which might mediate the interaction of circulatory, nervous and immune systems at acupoints. In normal rat LI4, serotonin-immunopositive MCs are present in subcutis and dermis, and their degranulation occurs in response to 3-min acupuncture [19]. Similar findings have been observed in ST36 of normal rats [98]. Additionally, local administration of serotonin in acupoint ST36 has an anti-nociceptive effect on mice suffering from CFA-induced ankle inflammatory pain or CCI neuropathic pain [90].

## 5. Interaction between MCs and Nerve Endings Ascends Acupuncture Signals

Acupuncture treatment on the skin is converted to neural signals and conducted from afferent neurons to the spinal cord and central nervous system [99]. The specificity of spatial contact between MCs and nerve has been demonstrated in vitro [100] and in vivo [101], including skin tissue [102]. The co-localization of MCs and nerve fibers, together with microvessels, is also present in rat acupoints [103]. In ankle AA rats, blocking peripheral nerves with lidocaine in the treated ST36 attenuates acupuncture analgesic effect, but does not markedly suppress the degranulation of MCs [104]. ST36 is innervated by the peroneal nerve, one of the branches of the sciatic nerve. In normal rats, needling ST36 increases the nerve discharge on sciatic nerve and dorsal root of lumbar 4 to 5, which is attenuated by preventing MC degranulation with CRO [105,106]. These findings suggest that needing-activated MCs are eventually involved in the analgesic effect via transmitting the biological signals to the nearby nerve endings, then ascending to the central nerve system.

Subcutaneous MC activation can drive neurogenic inflammation in skin [107]. SP and CGRP, together with histamine and serotonin, are expressed more in the LI4 acupoint of rats after acupuncture [19]. Actually, there is a mutual cause and effect relationship between neurogenic inflammation and activation of MCs. MC activation induces release of peptides from peripheral nerve terminals, including bradykinin, substance P (SP) and vasoactive intestinal peptide (VIP), gene-related peptide (CGRP) and amines, such as histamine and serotonin, which contribute to inflammation-mediated exacerbation of allergic reactions [108,109]. Furthermore, neuropeptides, such as CGRP, SP and VIP, also lead to the release of numerous inflammatory mediators from MCs through degranulation as a result of MC mediator-mediated nerve activation, which in turn enhances neurogenic inflammation and pain perception [108,110,111].

What role dose neurogenic inflammation play in the acupoints? When the body suffers from certain diseases, some corresponding acupoints become activated and sensitized, which is termed acupoint sensitization [112]. Neurogenic inflammation is one of the biological bases for acupoint sensitization due to dorsal root reflex or axon reflex [113,114]. For example, in rats, gastric mucosal injury results in neurogenic plasma extravasation in the skin of the related acupoints, together with more SP and CGRP [115]. Stimulation of sensitized acupoints presents a potential trend of generating a better clinical effect [112]. Acupoint sensitization is tightly associated with the activation of local MCs [92]. Additionally, SP and CGRP, together with histamine and serotonin, express more in the LI4 acupoints of rats treated by acupuncture [19].

Besides neurogenic inflammation, tissue injury-associated inflammation caused by needle piercing and manipulation also occurs in treated acupoints. Damage-associated molecules, high mobility HMGB1 and TLR4, and some inflammatory factors, e.g., TNF-αand IL-6, are upregulated in the treated ST36 by 2-min needling in normal rats [79]. Such localized inflammation is even supposed to play a vital role in a triggering acupuncture effect by initiating the systemic immune response.

## 6. Concluding Remarks and Further Directions

**Figure 20:**
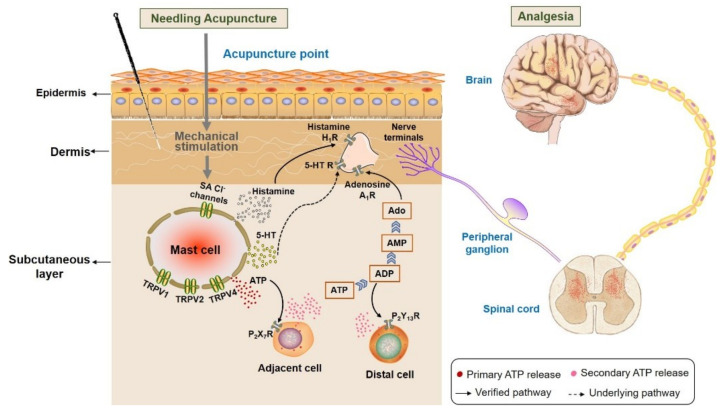
Activation of subcutaneous MCs triggers acupuncture analgesic effect. Needling acupuncture introduces physical stimuli to the treated acupoints. Subcutaneous MCs are activated to release various biological substances, including histamine, serotonin (5-HT) and ATP, through activating mechanosensitive channels. Subsequently, these substances or their downstream products bind to the corresponding receptors situated at local nerve terminals, or they mediate other cells to release ATP to amplify the local biochemical signals. Eventually, peripheral nerve endings ascend acupuncture signals to the peripheral ganglia, then to the spinal cord or upper spinal cord to generate analgesia.

With our review we wanted to demonstrate that the MC degranulation essentially contributes to triggering analgesia by acupuncture, which is summarized in Figure 20. Acupuncture stimuli include, in addition to the classical, mechanical stimulation by acupoint needling, acupoint stimulation by heat or laser light, which can all lead to analgesia. The activation of subcutaneous MC is based on activation of ion-selective channels that facilitate increases in intracellular Ca^2+^ activity, [Ca^2+^]_i_. Key roles play Ca^2+^-permeable TRPV that become activated by those stimuli, and also activation of stress-sensitive Cl^−^ channels contributes by membrane depolarization to enhanced [Ca^2+^]_i_. The increase in [Ca^2+^]_i_ is the basis for the exocytotic release of the mediators, including ATP, histamine and 5-HT; these mediators indicate the acupuncture signaling by activating sensory neurons. Important mediators of acupuncture signaling are also the metabolic ATP products by acting on various purinergic receptors. The high density of MCs in acupoints and their co-localization with afferent nerve endings suggests their functional interaction. It is now generally accepted that pain sensation in peripheral and CNS is modulated by extracellular nucleotides released by acupuncture [72]. Furthermore, MC-released histamine and serotonin play key roles in acupoint activation and analgesia by stimulating H1 and 5-HT 1 receptors, respectively. The stimulation of nerve ending by the activation of these receptors results in analgesic signaling via peripheral ganglia, the spinal cord, upper spinal cord and central nervous system.

Though we now have a detailed understanding about the role of subcutaneous MCs in triggering acupuncture-induced analgesia, single steps in the signaling pathway still need further clarification. Similarly, the role of the release of serotonin and stimulation of 5-HT1 receptors in analgesia needs further investigation.

## Data Availability

Not applicable.

## Abbreviations

AA	Adjuvant arthritis
acupoints	Acupuncture points
Ado	Adenosine
ACU	Acupuncture
ARL67156	6-*N*,*N*-Diethyl-β-γ-dibromomethylene-d-adenosine-5′-triphosphate
ATP	Adenosine triphosphate
CCI	Chronic constriction injury
CCPA	2-Chloro-*N*6-cyclopentyladenosine
CFA	Complete Freund’s adjuvant
CGRP	Calcitonin gene related peptide
CPM	Chlorprophenpyridamine maleate
CRO	Cromolyn
CTMC	Connective tissue mast cells
CWP	Ca^2+^ wave propagation
Ddo	Deamindiamine oxidase
DIDS	4,4′-Diisothiocyano-2,2′-stilbenedisulfonic acid
DMSO	Dimethyl sulfoxide
eATP	Extracellular ATP
ELISA	Enzyme linked immunosorbent assay
exo-ATP	Exogenous ATP
G_S_MT_X_4	M-theraphotoxin-Gr1a
HMGB1	High mobility group box 1 protein
HC067047	(2-Methyl-1-[3-(4-morpholinyl)propyl]-5-phenyl-*N*-[3-(trifluoromethyl) phenyl] -1H-pyrrole-3-carboxamide
HMC-1	Human Mast Cell Line 1
ICAM-1	Intercellular adhesion molecule-1
IR	Infrared
MCs	Mast cells
MCP-1	Monocyte chemoattractant protein-1
MMC	Mucosal mast cells
MRS2211	Calbiochem,2-[(2-Chloro-5-nitrophenyl)azo]-5-hydroxy-6-methyl-3-[(phosphonooxy)methyl]-4-pyridinecarboxaldehyde sodium salt
Pyrid.	2-pyridineethanamine dihydrochlorid
RBL-2H3	Rat alkaline leukemia mast cell line
RNA	Ribonucleic Acid
RT-PCR	Real-time polymerase chain reaction
SA	Stretch-activated
SACs	Stretch-activated chloride channels
SCF	Stem cell factor
SKF96365	1-[2-(4-methoxyphenyl)-2-[3-(4-methoxyphenyl)propoxy]ethyl]imidazole hydrochloride
SP	Substance P
TCM	Traditional Chinese medicine
TLR4	Toll-like receptor 4
TRPV	Transient receptor potential vanilloid
VIP	Vasoactive intestinal peptide
**List of Acupoints Name and Code**	
Feishu acupoint	BL13
Heding acupoint	EX-LE2
Yanglinquan acupoint	GB34
Hegu acupoint	LI4
Neiguan acupoint	PC6
Weishu acupoint	BL21
Zusanli acupoint	ST36

## References

[1] Gereau R.W., Sluka K.A., Maixner W., Savage S.R., Price T.J., Murinson B.B., Sullivan M.D., Fillingim R.B. (2014). A pain research agenda for the 21st century. J. Pain.

[2] Wang X., Yao W., Huang M., Zhang D., Xia Y., Xia Y. (2022). Signal Transduction in Acupoints. Advanced Acupuncture Research: From Bench to Bedside.

[3] Chon T.Y., Mallory M.J., Yang J., Bublitz S.E., Do A., Dorsher P.T. (2019). Laser acupuncture: A concise review. Med. Acupunct..

[4] Metcalfe D.D., Baram D., Mekori Y.A. (1997). Mast cells. Physiol. Rev..

[5] Kitamura Y., Go S., Hatanaka K. (1978). Decrease of mast cells in W/W*^v^* mice and their increase by bone marrow transplantation. Blood.

[6] Befus A.D., Bienenstock J., Denburg J.A. (1985). Mast cell differentiation and heterogeneity. Immunol. Today.

[7] Han J.S. (2004). Acupuncture and endorphins. Neurosci. Lett..

[8] Napadow V. (2018). When a white horse is a horse: Embracing the (obvious?) overlap between acupuncture and neuromodulation. J. Altern. Complement. Med..

[9] Shinbara H., Okubo M., Sumiya E., Fukuda F., Yano T., Kitade T. (2008). Effects of manual acupuncture with sparrow pecking on muscle blood flow of normal and denervated hindlimb in rats. Acupunct. Med..

[10] Li Y.M. (2021). In the search of mobile target for acupuncture: Why is mast cell?. Chin. Acupunct. Moxib..

[11] Li Y.M. (2019). Puzzles and hypotheses of acupuncture (II): From hypothesis to evidenced-based theory. Chin. J. Integr. Tradit. West. Med..

[12] Li Y.M. (2013). Perplexity and hypothesis in acupuncture research. Chin. J. Integr. Tradit. West. Med..

[13] Huo R.L., Ma S.X. (2016). Analysis of the Puzzle between Acupuncture Community and Acupuncture Clinical Trials. Chin. J. Integr. Tradit. West. Med..

[14] Song J.M. (1977). Mast cells and meridian phenomenon. Liaoning J. Tradit. Chin. Med..

[15] Liaoning College of Traditional Chinese Medicine, Department of Histology and Embryology, Department of Pathology (1980). Preliminary observation of mast cells in acupoint tissues. Liaoning J. Tradit. Chin. Med..

[16] Zhu Z.X., Xu R.M. (1990). Morphometric observation on the mast cells under the acupuncture meridian lines. Acupunct. Res..

[17] Zhu H., Wang X., Huang M., Jing Y., Zhang D., Ding G.H. (2017). Mast cell activation in the acupoint is important for the electroacupuncture effect against pituitrin-induced bradycardia in rabbits. Sci. Rep..

[18] Li Y. (2019). The neuroimmune basis of acupuncture: Correlation of cutaneous mast cell distribution with acupuncture systems in human. Am. J. Chin. Med..

[19] Wu M.L., Xu D.S., Bai W.Z., Cui J.J., Shu H.M., He W., Wang X.Y., Shi H., Su Y.S., Hu L. (2015). Local cutaneous nerve terminal and mast cell responses to manual acupuncture in acupoint LI4 area of the rats. J. Chem. Neuroanat..

[20] Yang H.W., Liu X.Y., Shen Z.F., Yao W., Gong X.B., Huang H.X., Ding G.H. (2018). An investigation of the distribution and location of mast cells affected by the stiffness of substrates as a mechanical niche. Int. J. Biol. Sci..

[21] Galli S.J., Tsai M., Wershil B.K. (1993). The c-kit receptor, stem-cell factor, and mast-cells. What each is teaching us about the others. Am. J. Pathol..

[22] Deshmane S.L., Kremlev S., Amini S., Sawaya B.E. (2009). Monocyte Chemoattractant Protein-1 (MCP-1): An Overview. J. Interferon Cytokine Res..

[23] Song X.J., Luo M.F., Jiang J., Zhang J.L. (2011). Effect of acupoint-injection of stem cell factor antibody on electroacupuncture induced changes of count and degranulation of subcutaneous mast cells in the local region in rats. Acupunct. Res..

[24] Song X.J., Luo M.F., Jiang J., Zhang J.L. (2013). Influences of injection of anti-MCP-1 in stomach acupoints on mastocyte distribution and function in rats. J. Beijing Univ. Tradit. Chin. Med..

[25] Song X.J., Luo M.F., Jiang J., Zhang J.L. (2014). Effects of electroacupuncture stimulation of “Zusanli” (ST 36) on the regional vascular intercellular adhesion molecule-1mRNA expression and mast cell activity in normal rats. Acupunct. Res..

[26] Zhang D., Ding G., Shen X., Yao W., Zhang Z., Zhang Y., Lin J., Gu Q. (2008). Role of mast cells in acupuncture effect: A pilot study. EXPLORE.

[27] Yu X., Ding G., Huang H., Lin J., Yao W., Zhan R. (2009). Role of collagen fibers in acupuncture analgesia therapy on rats. Connect. Tissue Res..

[28] Langevin H.M., Churchill D.L., Wu J., Badger G.J., Yandow J.A., Fox J.R., Krag M.H. (2002). Evidence of connective tissue involvement in acupuncture. FASEB J..

[29] Irani A.M., Craig S.S., DeBlois G., Elson C.O., Schechter N.M., Schwartz L.B. (1987). Deficiency of the tryptase-positive, chymase-negative mast cell type in gastrointestinal mucosa of patients with defective T lymphocyte function. J. Immunol..

[30] Lin J., Huang H., Ding G.H., Zhang D. (2007). Relationship between the function of mast cells and acupuncture analgesia in adjuvant arthritis rats. Acupunct. Res..

[31] Zhang D., Ding G., Shen X., Yao W., Zhang Z., Zhang L., Lin J., Gu Q. (2007). Influence of mast cell function on the analgesic effect of acupuncture of “Zusanli”(ST 36) in rats. Acupunct. Res..

[32] Huang M., Wang X., Xing B., Yang H., Sa Z., Zhang D., Yao W., Yin N., Xia Y., Ding G. (2018). Critical roles of TRPV2 channels, histamine H1 and adenosine A1 receptors in the initiation of acupoint signals for acupuncture analgesia. Sci. Rep..

[33] Wang X., Huang M., Yang H., Zhang D., Yao W., Xia Y., Ding G. (2020). Mast cell degranulation and adenosine release: Acupoint specificity for effect of electroacupuncture on pituitrin-induced acute heart bradycardia in rabbits. Evid.-Based Complement. Altern. Med..

[34] Cui X., Liu K., Xu D.D., Zhang Y.Y., He X., Liu H., Gao X.Y., Zhu B. (2018). Mast cell deficiency attenuates acupuncture analgesia for mechanical pain using c-kit gene mutant rats. J. Pain Res..

[35] Neher E. (1988). The influence of intracellular calcium concentration on degranulation of dialysed mast cells from rat peritoneum. J. Physiol..

[36] Nilius B., Flockerzi V., Barrett J.E. (2014). Mammalian transient receptor potential (TRP) cation channels. Preface. Handbook of Experimental Pharmacology.

[37] Bradding P., Okayama Y., Kambe N., Saito H. (2003). Ion channel gene expression in human lung, skin, and cord blood-derived mast cells. J. Leukoc. Biol..

[38] Zhang D., Spielmann A., Wang L., Ding G., Huang F., Gu Q., Schwarz W. (2012). Mast-cell degranulation induced by physical stimuli involves the activation of transient-receptor-potential channel TRPV2. Physiol. Res..

[39] Lin J.G., Hsieh C.L., Lin Y.W. (2015). Analgesic effect of electroacupuncture in a mouse fibromyalgia model: Roles of TRPV1, TRPV4, and pERK. PLoS ONE.

[40] Wu S.-Y., Chen W.-H., Hsieh C.-L., Lin Y.-W. (2014). Abundant expression and functional participation of TRPV1 at Zusanli acupoint (ST36) in mice: Mechanosensitive TRPV1 as an “acupuncture-responding channel”. Complement. Altern. Med..

[41] Chen W.H., Tzen J.T.C., Hsieh C.L., Chen Y.H., Lin T.J., Chen S.Y., Lin Y.W. (2012). Attenuation of TRPV1 and TRPV4 Expression and Function in Mouse Inflammatory Pain Models Using Electroacupuncture. Evid.-Based Complement. Altern. Med..

[42] Caterina M.J., Schumacher M.A., Tominaga M., Rosen T.A., Levine J.D., Julius D. (1997). The capsaicin receptor: A heat-activated ion channel in the pain pathway. Nature.

[43] Caterina M.J., Leffler A., Malmberg A.B., Martin W.J., Trafton J., Petersen-Zeitz K.R., Koltzenburg M., Basbaum A.I., Julius D. (2000). Impaired nociception and pain sensation in mice lacking the capsaicin receptor. Science.

[44] Roberts J.C., Davis J.B., Benham C.D. (2004). H-3 resiniferatoxin autoradiography in the CNS of wild-type and TRPV1 null mice defines TRPV1 (VR-1) protein distribution. Brain Res..

[45] Tominaga M., Caterina M.J., Malmberg A.B., Rosen T.A., Gilbert H., Skinner K., Raumann B.E., Basbaum A.I., Julius D. (1998). The cloned capsaicin receptor integrates multiple pain-producing stimuli. Neuron.

[46] Hu L., Wang L., Wei J., Ryszard G., Shen X., Wolfgang S. (2015). Heat induces adenosine triphosphate release from mast cells in vitro: A putative mechanism for moxibustion. J. Tradit. Chin. Med..

[47] Kanai Y., Hara T., Imai A., Sakakibara A. (2007). Differential involvement of TRPV1 receptors at the central and peripheral nerves in CFA-induced mechanical and thermal hyperalgesia. J. Pharm. Pharmacol..

[48] Gu Q., Wang L., Huang F., Schwarz W. (2012). Stimulation of TRPV1 by green laser light. Evid.-Based Complement. Altern. Med..

[49] Gunthorpe M.J., Benham C.D., Randall A., Davis J.B. (2002). The diversity in the vanilloid (TRPV) receptor family of ion channels. Trends Pharmacol. Sci..

[50] Numazaki M., Tominaga M. (2004). Nociception and TRP channels. Curr. Drug Targets—CNS Neurol. Disord..

[51] Stokes A.J., Shimoda L.M.N., Koblan-Huberson M., Adra C.N., Turner H. (2004). A TRPV2-PKA signaling module for transduction of physical stimuli in mast cells. J. Exp. Med..

[52] Benham C.D., Gunthorpe M.J., Davis J.B. (2003). TRPV channels as temperature sensors. Cell Calcium.

[53] O’Neil R.G., Heller S. (2005). The mechanosensitive nature of TRPV channels. Pflug. Arch..

[54] Zheng Y., Zuo W., Shen D., Cui K., Huang M., Zhang D., Shen X., Wang L. (2021). Mechanosensitive TRPV4 channel-induced extracellular ATP accumulation at the acupoint mediates acupuncture analgesia of ankle arthritis in rats. Life.

[55] Alessandri-Haber N., Yeh J.J., Boyd A.E., Parada C.A., Chen X., Reichling D.B., Levine J.D. (2003). Hypotonicity Induces TRPV4-mediated nociception in rat. Neuron.

[56] Alessandri-Haber N., Joseph E., Dina O.A., Liedtke W., Levine J.D. (2005). TRPV4 mediates pain-related behavior induced by mild hypertonic stimuli in the presence of inflammatory mediator. Pain.

[57] Dias F.C., Alves V.S., Matias D.O., Figueiredo C.P., Miranda A.L.P., Passos G.F., Costa R. (2019). The selective TRPV4 channel antagonist HC-067047 attenuates mechanical allodynia in diabetic mice. Eur. J. Pharmacol..

[58] Wang L., Zhang D., Schwarz W. (2014). TRPV Channels in Mast Cells as a Target for Low-Level-Laser Therapy. Cells.

[59] Yang W.Z., Chen J.Y., Yu J.T., Zhou L.W. (2007). Effects of low power laser irradiation on intracellular calcium and histamine release in RBL-2H3 mast cells. Photochem. Photobiol..

[60] Wang L., Schwarz W. (2012). Activation of mast cells by acupuncture stimuli. Forum Immunopathol. Dis. Ther..

[61] Mascarenhas N.L., Wang Z., Chang Y.L., Di Nardo A. (2017). TRPV4 mediates mast cell activation in cathelicidin-induced rosacea inflammation. J. Investig. Dermatol..

[62] Wang L., Ding G., Gu Q., Schwarz W. (2010). Single-channel properties of a stretch-sensitive chloride channel in the human mast cell line HMC-1. Eur. Biophys. J..

[63] Mikolajewicz N., Mohammed A., Morris M., Komarova S.V. (2018). Mechanically stimulated ATP release from mammalian cells: Systematic review and meta-analysis. J. Cell Sci..

[64] Lazarowski E.R. (2012). Vesicular and conductive mechanisms of nucleotide release. Purinergic Signal..

[65] Zimmermann H. (2016). Extracellular ATP and other nucleotides—Ubiquitous triggers of intercellular messenger release. Purinergic Signal..

[66] Burnstock G. (2009). Acupuncture: A novel hypothesis for the involvement of purinergic signalling. Med. Hypotheses.

[67] Tang Y., Yin H., Rubini P., Illes P. (2016). Acupuncture-induced analgesia: A neurobiological basis in purinergic signaling. Neuroscientist.

[68] Takano T., Chen X., Luo F., Fujita T., Ren Z., Goldman N., Zhao Y., Markman J.D., Nedergaard M. (2012). Traditional acupuncture triggers a local increase in adenosine in human subjects. J. Pain.

[69] Goldman N., Chen M., Fujita T., Xu Q., Peng W., Liu W., Jensen T.K., Pei Y., Wang F., Han X. (2010). Adenosine A1 receptors mediate local anti-nociceptive effects of acupuncture. Nat. Neurosci..

[70] Marquardt D.L., Gruber H.E., Wasserman S.I. (1984). Adenosine release from stimulated mast cells. Proc. Natl. Acad. Sci. USA.

[71] Ding G., Zhang D., Huang M., Wang L., Yao W., Xia Y., Ding G., Wu G. (2012). The function of collagen and mast cells at acupoints. Current Research in Acupuncture.

[72] Tang Y., Yin H., Liu J., Rubini P., Illes P. (2019). P2X receptors and acupuncture analgesia. Brain Res. Bull..

[73] Wang L., Sikora J., Hu L., Shen X., Grygorczyk R., Schwarz W. (2013). ATP release from mast cells by physical stimulation: A putative early step in activation of acupuncture points. Evid.-Based Complement. Altern. Med..

[74] Zuo W.M., Li Y.J., Cui K.Y., Shen D., Zhang D., Zhen Y.W., Huang M., Wu Y., Shen X.Y., Wang L.N. (2022). The real-time detection of acupuncture-induced extracellular ATP mobilization in acupoints and exploration its role in acupuncture analgesia. Purinergic Signal..

[75] Miyamoto T., Mochizuki T., Nakagomi H., Kira S., Watanabe M., Takayama Y., Suzuki Y., Koizumi S., Takeda M., Tominaga M. (2014). Functional role for Piezo1 in stretch-evoked Ca^2+^ influx and ATP release in urothelial cell cultures. J. Biol. Chem..

[76] Cinar E., Zhou S., DeCourcey J., Wang Y., Waugh R.E., Wan J. (2015). Piezo1 regulates mechanotransductive release of ATP from human RBCs. Proc. Natl. Acad. Sci. USA.

[77] Wang S., Chennupati R., Kaur H., Iring A., Wettschureck N., Offermanns S. (2016). Endothelial cation channel PIEZO1 controls blood pressure by mediating flow-induced ATP release. J. Clin. Investig..

[78] Egbuniwe O., Grover S., Duggal A.K., Mavroudis A., Yazdi M., Renton T., Di Silvio L., Grant A.D. (2014). TRPA1 and TRPV4 activation in human odontoblasts stimulates ATP release. J. Dent. Res..

[79] Qiang X.I., Cui R., Jin G., Guo Y.M. (2015). Preliminary research on the role of TLR4 at acupoints in the inflammatory reaction produced by acupuncture. Tianjin J. Tradit. Chin. Med..

[80] Burnstock G., Barrett J.E. (2016). Purinergic mechanisms and pain. Pharmacological Mechanisms and the Modulation of Pain.

[81] Hamilton S.G., James W., Anupam B., Jane W., Mcmahon S.B. (2000). ATP in human skin elicits a dose-related pain response which is potentiated under conditions of hyperalgesia. Brain.

[82] Hamilton S.G., Wade A., Mcmahon S.B. (2010). The effects of inflammation and inflammatory mediators on nociceptive behaviour induced by ATP analogues in the rat. Br. J. Pharmacol..

[83] Shen D., Shen X., Schwarz W., Grygorczyk R., Wang L. (2020). P2Y_13_ and P2X_7_ receptors modulate mechanically induced adenosine triphosphate release from mast cells. Exp. Derm..

[84] Zimmermann H., Zebisch M., Strater N. (2012). Cellular function and molecular structure of ecto-nucleotidases. Purinergic Signal..

[85] Li G., Liang J.M., Li P.W., Yao X., Pei P.Z., Li W., He Q.H., Yang X., Chan Q.C., Cheung P.Y. (2011). Physiology and cell biology of acupuncture observed in calcium signaling activated by acoustic shear wave. Pflug. Arch..

[86] Ohsaki A., Tanuma S., Tsukimoto M. (2018). TRPV4 channel-regulated ATP release contributes to gamma-irradiation-induced production of IL-6 and IL-8 in epidermal keratinocytes. Biol. Pharm. Bull..

[87] Ho T.C., Horn N.A., Huynh T., Kelava L., Lansman J.B. (2012). Evidence TRPV4 contributes to mechanosensitive ion channels in mouse skeletal muscle fibers. Channels.

[88] Schneider E., Rolli-Derkinderen M., Arock M., Dy M. (2002). Trends in histamine research: New functions during immune responses and hematopoiesis. Trends Immunol..

[89] Huang M., Xie Y.Y., Ding G.H. (2010). Acupoint-injection of histamine induced analgesic effect in acute adjuvant-induced-arthritis rats. Acupunct. Res..

[90] Vieira J.S., Toreti J.A., de Carvalho R.C., de Araujo J.E., Silva M.L., Silva I.R.T. (2018). Analgesic effects elicited by neuroactive mediators injected into the ST 36 acupuncture point on inflammatory and neuropathic pain in mice. J. Acupunct. Meridian Stud..

[91] Parsons M.E., Ganellin C.R. (2006). Histamine and its receptors. Br. J. Pharmacol..

[92] Ding N., Jiang J., Qin P., Wang Q., Hu J., Li Z. (2018). Mast cells are important regulator of acupoint sensitization via the secretion of tryptase, 5-hydroxytryptamine, and histamine. PLoS ONE.

[93] Zhang G.S., Qiu R.R., Pan J., Zhang J., Zhang C., Wang C.X., Yang R.D. (2020). Effect of moxibustion on respiratory function and cutaneous histamine and neuropeptide contents of “Feishu” (BL13) in asthmatic rats. Acupunct. Res..

[94] Chen H., Dong Sheng X.U., Yang J.S., Bai W.Z., Yan P.H., Wang Y.Y. (2018). Effect of moxibustion on the expressions of 5-hydroxytryptamine and histamine in skin tissue at yanglingquan (GB34). Shanghai J. Acupunct. Moxib..

[95] Wingren U., Wasteson A., Enerback L. (1983). Storage and turnover of histamine, 5-hydroxytryptamine and heparin in rat peritoneal mast cells in vivo. Int. Arch. Allergy Appl. Immunol..

[96] Theoharides T.C., Bondy P.K., Tsakalos N.D., Askenase P.W. (1982). Differential release of serotonin and histamine from mast cells. Nature.

[97] Tore F., Tuncel N. (2009). Mast Cells: Target and Source of Neuropeptides. Curr. Pharm. Des..

[98] Dimitrov N., Atanasova D., Tomov N., Sivrev D., Lazarov N. (2017). Acupuncture causes serotonin release by mast cells. Rom. J. Morphol. Embryol..

[99] Zhao Z.Q. (2008). Neural mechanism underlying acupuncture analgesia. Prog. Neurobiol..

[100] Blennerhassett M.G., Tomioka M., Bienenstock J. (1991). Formation of contacts between mast cells and sympathetic neurons in vitro. Cell Tissue Res..

[101] Arizono N., Matsuda S., Hattori T., Kojima Y., Maeda T., Galli S.J. (1990). Anatomical variation in mast cell nerve associations in the rat small intestine, heart, lung, and skin. Similarities of distances between neural processes and mast cells, eosinophils, or plasma cells in the jejunal lamina propria. Lab. Investig..

[102] Bauer O., Razin E. (2000). Mast cell-nerve interactions. Physiology.

[103] Mingfu L., Xiaotong D., Xiaojing S., Jin J., Jinling Z., Ying H. (2013). Study on the dynamic compound structure composed of mast cells, blood vessels, and nerves in rat acupoint. Evid.-Based Complement. Altern. Med..

[104] Huang H., Zhan R., Yu X.J., Zhang D., Li W.-M., Ding G.H. (2009). Effects of acupoint-nerve block on mast cell activity, manual acupuncture- and electroacupuncture-induced analgesia in adjuvant arthritis rats. Acupunct. Res..

[105] Sa Z.Y., Huang M., Zhang D., Ding G.H. (2013). Relationship between regional mast cell activity and peripheral nerve discharges during manual acupuncture stimulation of “Zusanli” (ST 36). Acupunct. Res..

[106] Yin N., Yang H.W., Yao W., Xia Y., Ding G.H. (2018). Mast cells and nerve signal conduction in acupuncture. Evid.-Based Complement. Altern. Med..

[107] Siiskonen H., Harvima I. (2019). Mast cells and sensory nerves contribute to neurogenic inflammation and pruritus in chronic skin inflammation. Front. Cell. Neurosci..

[108] Kilinc E., Firat T., Tore F., Kiyan A., Kukner A., Tuncel N. (2015). Vasoactive intestinal peptide modulates c-Fos activity in the trigeminal nucleus and dura mater mast cells in sympathectomized rats. J. Neurosci. Res..

[109] Kilinc E., Guerrero-Toro C., Zakharov A., Vitale C., Gubert-Olive M., Koroleva K., Timonina A., Luz L.L., Shelukhina I., Giniatullina R. (2017). Serotonergic mechanisms of trigeminal meningeal nociception: Implications for migraine pain. Neuropharmacology.

[110] Dagistan Y., Kilinc E., Balci C.N. (2019). Cervical sympathectomy modulates the neurogenic inflammatory neuropeptides following experimental subarachnoid hemorrhage in rats. Brain Res..

[111] Kilinc E., Gunes H. (2019). Modulatory effects of neuropeptides on pentylenetetrazol-induced epileptic seizures and neuroinflammation in rats. Rev. Assoc. Med. Bras..

[112] Tan H., Tumilty S., Chapple C., Liu L., McDonough S., Yin H., Yu S., Baxter G.D. (2019). Understanding acupoint sensitization: A narrative review on phenomena, potential mechanism, and clinical application. Evid.-Based Complement. Altern. Med..

[113] Kim D.H., Ryu Y., Hahm D.H., Sohn B.Y., Shim I., Kwon O.S., Chang S., Gwak Y.S., Kim M.S., Kim J.H. (2017). Acupuncture points can be identified as cutaneous neurogenic inflammatory spots. Sci. Rep..

[114] Zhang M., Guo H., Ma Y., Xu F., Bai F., Liang S., Hu H., Wang Q., Deng J., Dong H. (2019). Acupoint sensitization is associated with increased excitability and hyperpolarization-activated current (I_h_) in C- but not Adelta-type neurons. Neuroscience.

[115] He W., Wang X.Y., Shi H., Bai W.Z., Cheng B., Su Y.S., Yu X.C., Jing X.H., Zhu B. (2017). Cutaneous neurogenic inflammation in the sensitized acupoints induced by gastric mucosal injury in rats. BMC Complement. Altern. Med..

